# CD8^+^ T cell responses recognizing immunodominant *Chlamydia* antigens fail to protect against infection

**DOI:** 10.1016/j.isci.2026.115242

**Published:** 2026-03-05

**Authors:** Safia Guleed, Nina Dieu Nhien Tran Nguyen, Sharmila Subratheepam, Kristoffer Mazanti Melchiors, Anja Weinreich Olsen, Matias Ciancaglini, Anna Lena Kastner, Emanuele Nolfi, Daniel Pinschewer, Frank Follmann, Jan Pravsgaard Christensen, Alice Sijts, Jes Dietrich

**Affiliations:** 1 Department of Infectious Disease Immunology, Center for Vaccine Research, Statens Serum Institut, Copenhagen, Denmark; 2 Department of Immunology & Microbiology, University of Copenhagen, Copenhagen, Denmark; 3 Division of Experimental Virology, Department of Biomedicine, University of Basel, Basel, Switzerland; 4 Department of Infectious Disease and Immunology, Utrecht University, Utrecht, the Netherlands; 5 Leibniz Institute for Immunotherapy, Regensburg, Germany

**Keywords:** Biological sciences, Immunology

## Abstract

*Chlamydia trachomatis* (C.t.) causes the most common bacterial sexually transmitted infection, yet no licensed vaccine exists. CD4^+^ T cells are known to protect, while the role of CD8^+^ T cells is unclear. This study examined whether CD8^+^ T cells targeting five immunodominant C.t. proteins could protect against infection. Using an *in silico* approach, we identified six novel CD8^+^ T cell epitopes from ArtJ, GroES, IncA, OmpH, and major outer membrane protein (MOMP). These epitopes elicited C.t.-specific CD8^+^ T cells when delivered with the cationic adjuvant formulation 09b (CAF09b) liposomal adjuvant or a lymphocytic choriomeningitis virus (LCMV) vector expressing the MOMP-based CTH522 protein. After C.t. challenge, vaccine-induced CD8^+^ T cells rapidly accumulated in the genital tract, became resident after bacterial clearance, produced effector cytokines, and showed strong cytotoxicity. However, neither single nor combined CD8^+^ T cell responses conferred protection, whereas a CD4^+^ T cell-inducing CTH522-based vaccine did. This implies that vaccine-induced CD8^+^ T cells play a minor or no role in protection against C.t.

## Highlights

• Six novel CD8^+^ T cell epitopes identified from immunodominant *Chlamydia trachomatis* proteins
• Vaccination with the epitopes in the adjuvant CAF09b generated cytotoxic CD8^+^ T cells
• Vaccine-induced CD8^+^ T cells were efficiently recruited to the site of infection
• In contrast to a CD4^+^ vaccine, vaccine-induced CD8^+^ T cells gave no protection

## Graphical abstract

**Figure:**
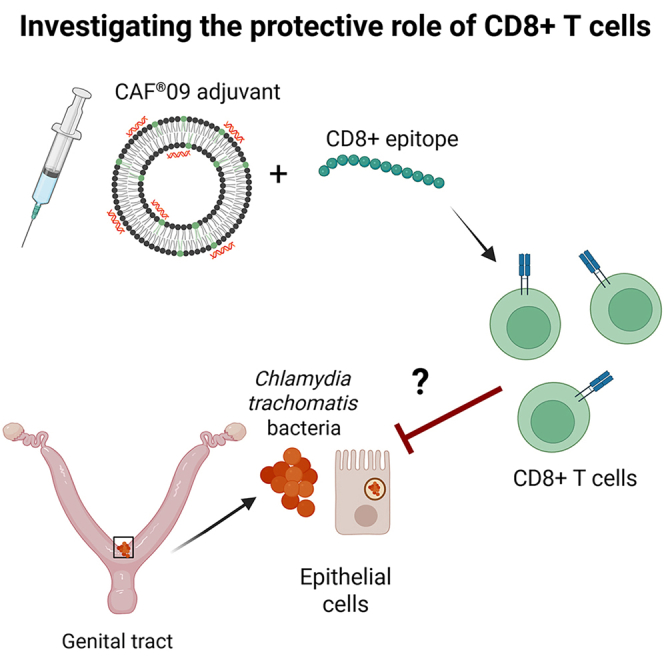

## Introduction

*Chlamydia trachomatis* (C.t.) is the obligate intracellular bacterium responsible for the most prevalent bacterial sexually transmitted disease globally. It accounts for an estimated 128 million new cases annually, with a substantial proportion of asymptomatic infections.^1^^,^^2^ Untreated *Chlamydia* infections in women can result in serious complications such as pelvic inflammatory disease, ectopic pregnancy, and infertility, posing a significant public health challenge.^3^^,^^4^^,^^5^

Current interventions rely on antibiotic treatment, which, while effective against infection, does not prevent repeated infections.^2^ Despite the long-standing international priority to develop a vaccine, protective immunity against the bacterium is still not fully resolved,^6^^,^^7^ likely due to its complex biphasic life cycle.^8^^,^^9^ Although it is generally accepted that CD4^+^ T cells and antibodies play an essential role in protective immunity against C.t. infection,^10^^,^^11^^,^^12^^,^^13^^,^^14^^,^^15^ the role of CD8^+^ T cells is less established and remains a subject of debate.

CD8^+^ T cell responses are the primary facilitators of immunity against viral infections.^16^ During genital tract (GT) infections with human immunodeficiency virus (HIV) and herpes simplex virus (HSV), CD8^+^ T cells play a crucial role in limiting the viral load.^17^^,^^18^ Furthermore, in HSV-2 infections, it has been demonstrated that CD8^+^ T cells capable of IFN-γ production and cytolysis are required for clearance of infection.^18^ Although it is reasonable to speculate that CD8^+^ T cells may also play a role against intracellular bacterial pathogens such as C.t., *Mycobacterium tuberculosis*, or *Salmonella enterica*, the protective role of CD8^+^ T cells against these pathogens is not clear, in contrast to the established role of CD4^+^ T cells.^19^^,^^20^^,^^21^^,^^22^ Concerning C.t., the primary target cell is epithelial cells, and some C.t. antigens have been shown to escape to the cytosol and be presented on MHC-I to CD8^+^ T cells.^23^^,^^24^

Most studies examining the role of CD8^+^ T cells in C.t. infection have predominantly focused on infection-induced CD8^+^ T cells that were shown to have no protective role or even a negative impact on both protection and the development of harmful pathology.^25^^,^^26^^,^^27^^,^^28^^,^^29^^,^^30^ In contrast, a recent study in humans reported that the presence of IFN-γ^+^ CD8^+^ T cells correlated with lower chlamydia bacterial load in reinfected women, and vaccine studies in non-human primates (NHPs) indicated a protective role of CD8^+^ T cells during a C.t. infection.^31^^,^^32^^,^^33^ Taken together, whether CD8^+^ T cells are required for protection, and consequently should be given priority in future vaccine strategies, is still not fully resolved.

To date, only three CD8^+^ T cell epitopes have been identified in the context of murine C.t. infection.^24^^,^^34^^,^^35^ Among these, the protective potential has been investigated for only one epitope, which showed partial protection against systemic C.t. infection when induced with recombinant vaccinia virus.^24^ In the present study, we identified a total of six novel CD8^+^ T cell epitopes. We successfully generated CD8^+^ T cells against all the epitopes using either a novel adjuvant system “cationic adjuvant formulation 09b” (CAF09b), which we developed to cross-present antigens on MHC class I due to inclusion of the TLR3 agonist poly(I:C),^36^ or a recombinant lymphocytic choriomeningitis virus (rLCMV) vector.^37^ Here, we investigate the functional capabilities of these CD8^+^ T cells and whether their recruitment to an infected GT provided protection against infection.

## Results

### CD8^+^ T cells are induced after a genital infection

**Figure 1:**
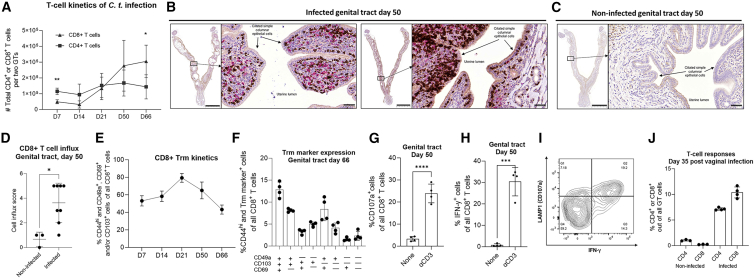
CD8^+^ T cells are induced after genital infection with C.t. and express tissue-resident memory markers (A–J) Eight to ten weeks old female B6C3F1 mice were subjected to transcervical infection (1 × 10^3^ IFUs) (A) Absolute T cell numbers of CD4^+^ and CD8^+^ T cells (mean ± SD, unpaired *t* test) in the GT tissue were determined by flow cytometry at days 7, 14, 21 (*n* = 16 biological replicates, pooled pairwise), 50, and 66 post infection (*n* = 8 biological replicates, pooled pairwise). (B and C) Immunohistochemical staining of CD8 (brown chromogen) and CD4 (red chromogen) in the genital tract uterine horns day 50 post challenge. Representative image of uterine horn sections from (B) two infected animals (*n* = 12 biological replicates) and (C) a non-infected animal (*n* = 3 biological replicates), with 0.3× magnification (left, scale bars: 5,000 μm) and 20× magnification (right, scale bars: 50 μm). (D) All animals were assigned a CD8^+^ T cell influx score (mean ± SD, unpaired *t* test), based on the degree of CD8^+^ T cell infiltration in the uterine tissue. (E) %CD8^+^ Trm cells (CD44^hi^ and CD49a^+^, CD69^+^ and/or CD103^+^) of all CD8^+^ T cells (mean ± SD) throughout infection. (F) Combination of %Trm marker expression of CD8^+^ T cells (mean ± SD) present at day 66 post infection. (G and H) (G) %Expression of surface CD107a (LAMP1) and (H) intracellular IFN-γ of CD8^+^ T cells (mean ± SD, unpaired *t* test) in the GT upon αCD3e stimulation at day 50 post infection. (I) Representative contour plot of CD107^+^ and IFN-γ^+^-CD8^+^ T cells in the GT. (J) Eight to ten weeks old female B6C3F1 mice were infected vaginally with 5 × 10^4^ IFUs of C.t. SvD. At day 35 post infection, %CD4^+^ and CD8^+^ T cell levels in the GT were determined by flow cytometry (*n* = 8 biological replicates, pooled pairwise, mean ± SD). IFUs, inclusion forming units; GT, genital tract. Trm, tissue-resident memory T cell. Statistical significance represented as ∗*p* < 0.05, ∗∗*p* < 0.01, ∗∗∗*p* < 0.001, ∗∗∗∗*p* < 0.0001.

Since an important part of our study was to discover infection-induced CD8^+^ T cell epitopes, it was first important to determine to what degree CD8^+^ T cells were induced by an infection in our mouse model. Female B6C3F1 mice were subjected to a transcervical (TC) infection with C.t. serovar D (SvD), and CD8^+^ T cell responses in the GT over time were measured by flow cytometry analysis. To distinguish leukocytes in the GT tissue from circulating leukocytes, mice were subjected to *in vivo* intravascular staining by intravenous (i.v.) administration of FITC-conjugated anti-mouse CD45.2 monoclonal antibody (mAb) (i.v. CD45) prior to euthanasia.^38^ At day 7 post C.t. challenge, we observed higher numbers of CD4^+^ T cells, compared to CD8^+^ T cells, in the GT tissue (Figure 1A). However, in the later stages of infection, the CD8^+^ T cell numbers surpassed CD4^+^ T cell numbers (Figure 1A). This CD8^+^ T cell dominance was further confirmed by immunohistochemical (IHC) staining of CD8^+^ and CD4^+^ in the upper GT at day 50 post infection, visualized as brown (CD8^+^ T cells) and red chromogen stains (CD4^+^ T cells) (Figures 1B and 1C). The CD8^+^ T cells exhibited a distinct tissue localization in the GT and were positioned in close contact with the uterine epithelial layer (Figure 1B). All animals were assigned a score, based on the degree of CD8^+^ T cell infiltration. There was a significantly higher CD8^+^ T cell influx in infected mice compared to age-matched non-infected mice (Figure 1D). Recruited CD8^+^ T cells expressed tissue-resident memory T cell (Trm) markers CD69, CD49a, and CD103, involved in tissue retention, migration, and positioning of CD8^+^ T cells.^39^^,^^40^ One week after infection, approximately 53% of CD8^+^ T cells within the tissue were CD44^hi^ and expressed CD69, CD49a, and/or CD103 (Figure 1E). The CD44^hi^ and CD69^+^, CD49a^+^, and/or CD103^+^ CD8^+^ T cells remained throughout the course of infection from day 7 to day 66 post infection (Figure 1E). By day 66 post infection, 13% of CD8^+^ T cells co-expressed all three Trm markers, and 8% co-expressed CD49a and CD103. Other combinations of two Trm markers, as well as cells expressing only a single Trm marker, each represented less than 5% of CD8^+^ T cells present in the GT. Only 2.34% of the GT CD8^+^ T cells lacked expression of the Trm markers but were still CD44^hi^ (Figure 1F). Together with their persistence at late time points and absence of the i.v. CD45.2 labeling, these data show that the majority of antigen-experienced CD8^+^ T cells (CD44^hi^) exhibit a Trm phenotype.

Following *in vitro* stimulation with plate-bound anti-mouse CD3e mAb, 24% and 30% of GT CD8^+^ T cells were positive for degranulation marker CD107a^41^ and IFN-γ, respectively (Figures 1G–1I). Finally, as observed with a TC infection, a vaginal infection with C.t. also led to increased levels of CD8^+^ T cells in the GT more than 1 month following infection with C.t. (Figure 1J).

In summary, following a TC or vaginal infection with C.t., functional CD8^+^ T cells were induced and recruited to the GT, implying that these models can be used to discover novel CD8^+^ T cell epitopes.

### Discovery of novel CD8^+^ T cell epitopes in four C.t. proteins

To investigate the vaccine potential of C.t.-specific CD8^+^ T cells, we developed a strategy to identify novel CD8^+^ T cell epitopes (Figure S1). In brief, the strategy initially entailed the selection of 10 immunodominant C.t. antigens, highly recognized in two or more of the studies examining antibody and/or T cell responses against chlamydial proteins from humans or from animal infection models (Table 1). Second, we conducted *in silico*-based predictions of CD8^+^ T cell epitopes restricted to H2-K^b^, H2-K^k^, and H2-D^b^ molecules utilizing the online NetMHCpan-4.1 tool for subsequent selection of peptides classified as strong binders.^42^ A total of 20 peptide candidates (9-mers) were selected and synthesized (Table S1). Finally, all epitopes were evaluated for their immunogenicity in our mouse model, and those exhibiting immunogenic properties were further analyzed for their capacity to induce IFN-γ and TNF-α expression in CD8^+^ T cells from C. t.-infected (non-vaccinated) mice to determine whether they also represented infection-induced epitopes.

**Table 1:** Selected immunodominant proteins from infection with *C. trachomatis* in humans and experimental animal models

Locus tag	Protein description (Uniprot)	Gene name (Uniprot)	Length (aa)	Reference
CT110	chaperonin GroEL 60 kD	GroEL, GroL	544	Follmann et al.^43^, Finco et al.^44^, Rodgers et al.^45^, Teng et al.^46^, Patton et al.^47^, Hufnagel et al.^48^, Randall et al.^49^
CT111	co-chaperonin GroES 10 kD	GroES, GroS	102	Teng et al.^46^, Patton et al.^47^
CT119	inclusion membrane protein A	IncA	273	Wang et al.^50^, Finco et al.^44^, Rodgers et al.^45^, Teng et al.^46^, Patton et al.^47^, Randall et al.^49^
CT242	Skp-like protein	OmpH	173	Finco et al.^44^, Patton et al.^47^, Hufnagel et al.^48^, Lu et al.^51^, Randall et al.^49^
CT381	probable ABC transporter arginine-binding protein	ArtJ	257	Wang et al.^50^, Finco et al.^44^, Rodgers et al.^45^, Teng et al.^46^, Randall et al.^49^
CT442	sulfur-rich protein	CrpA, srp	150	Wang et al.^50^, Rodgers et al.^45^, Teng et al.^46^, Patton et al.^47^, Hufnagel et al.^48^
CT443	large cysteine-rich periplasmic protein (CRP)	OmcB	547	Follmann et al.^43^, Wang et al.^50^, Finco et al.^44^, Rodgers et al.^45^, Teng et al.^46^, Patton et al.^47^, Lu et al.^51^, Randall et al.^49^
CT541	peptidyl-prolyl *cis*-*trans* isomerase Mip	Mip	243	Finco et al.^44^, Teng et al.^46^, Patton et al.^47^, Lu et al.^51^, Randall et al.^49^, Hufnagel et al.^48^
CT603	thio-specific antioxidant (TSA) peroxidase	AhpC	195	Rodgers et al.^45^, Olsen et al.^52^
CT823	probable periplasmic serine endoprotease DegP-like	HtrA	497	Finco et al.^44^, Rodgers et al.^45^, Hokynar et al.^53^

To generate vaccine-induced CD8^+^ T cells, we used the vaccine adjuvant CAF09b, a liposomal delivery system comprising dimethyldioctadecylammonium bromide (DDA), the C-type lectin receptor agonist monomycoloyl glycerol (MMG), and the TLR3 agonist poly(I:C). CAF09b is specifically recognized for its capacity to induce robust CD8^+^ T cell responses.^54^^,^^55^^,^^56^

**Figure 2:**
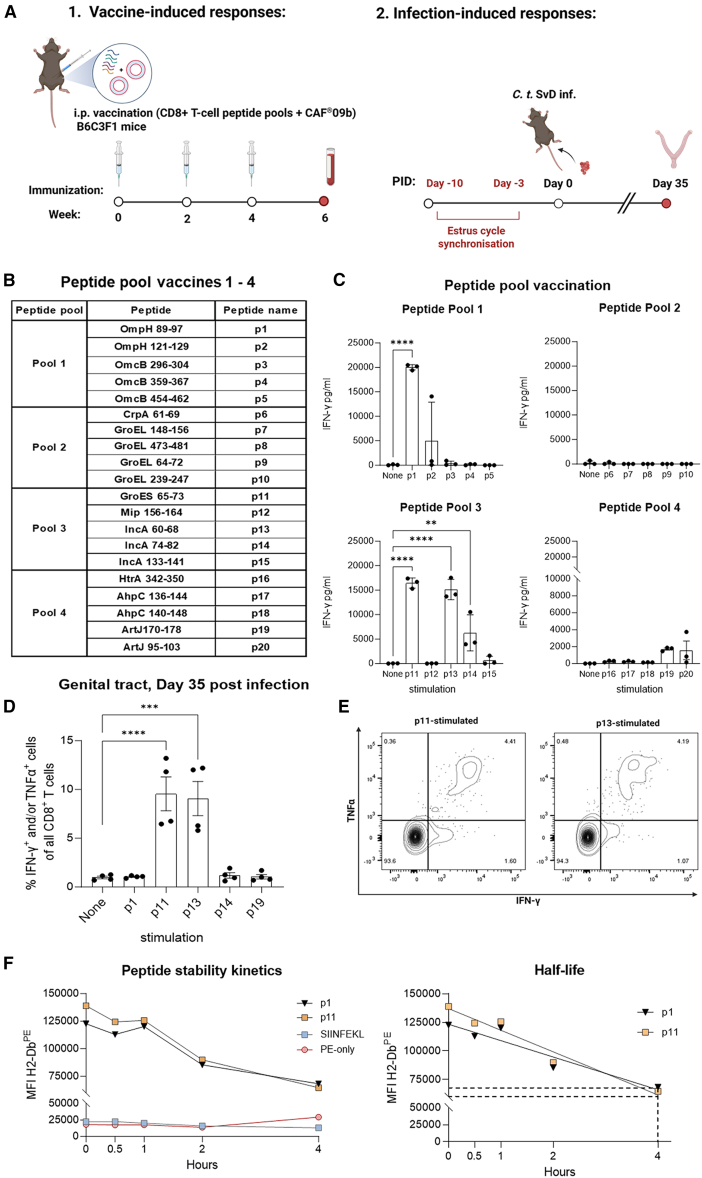
Identification of *C. trachomatis*-specific CD8^+^ T cell epitopes (A) Experimental design of immunogenicity testing of CD8^+^ T cell peptide candidates in mouse model. 1. Induction of peptide-specific responses with CAF09b vaccination. 2. Analysis of activated GT CD8^+^ T cells after infection for the identification of immunodominant C.t. epitopes. (B) Table showing the selected CD8^+^ T cell candidates for peptide pool immunizations, predicted from immunodominant C.t. protein antigens (Table S1). (C) Groups of female B6C3F1 mice were vaccinated three times intraperitoneally with the different peptide pools adjuvanted in CAF09b (*n* = 16, all mice pooled and data points represent replicates). Blood was sampled from the mice 10 days post last immunization. PBMCs were isolated and stimulated with indicated peptides for 72 h, whereas IFN-γ secretion was measured in the supernatant by ELISA (mean ± SD, one-way ANOVA followed by Dunnett’s multiple comparison test). (D) GT tissue from female B6C3F1 mice subjected to vaginal C.t. challenge (5 × 10^4^ IFUs) was re-stimulated with CD8^+^ T cell peptide candidates at day 35 post infection (*n* = 16 biological replicates, all mice pooled and data points represent technical replicates). Frequency of intracellular expression of TNF-α and/or IFN-γ by CD8^+^ T cells was measured by flow cytometry (mean ± SD, one-way ANOVA followed by Dunnett’s multiple comparison test). (E) Representative flow cytometry contour plot of Cyt^+^ CD8^+^ T cells in the genital tract upon p11 or p13 stimulation. (F) Peptide-binding stability assay to H2-D^b^ molecule (left) and graphic estimation of half-time (right). Surface expression of H2-D^b^ molecules in the present peptides or controls was measured after 30 min, 1 h, 2 h, and 4 h of incubation and analyzed with flow cytometry analysis. Values are shown as median fluorescence intensity. PID, post infection day; i.p., intraperitoneal; IFUs, inclusion forming units; GT, genital tract; Cyt^+^, cytokine positive; ELISA, enzyme-linked immunosorbent assay. Statistical significance represented as ∗∗*p* < 0.01, ∗∗∗*p* < 0.001, ∗∗∗∗*p* < 0.0001.

Groups of B6CF1 mice were immunized three times with pools of *Chlamydia* peptides in CAF09b, and T cell responses against the individual epitopes were assessed with peripheral blood mononuclear cells (PBMCs) 2 weeks after the last immunization (Figures 2A and 2B). When measuring IFN-γ secretion from stimulated PBMCs, significant C.t.-specific T cell responses were observed with 4 out of the 20 predicted epitopes, p1, p11, p13, and p14 (OmpH_89–97_, GroES_65–73_, IncA_60–68_, and IncA_74–82_) (Figure 2C). A trend was observed for p19 (ArtJ_170–178_), and flow cytometry analysis in subsequent studies confirmed the immunogenic properties of p19 and that all peptide-specific responses were restricted to CD8^+^ T cells (Figures S2A–S2B).

After confirming their immunogenic properties following immunization, we further examined whether any of the epitopes were induced by infection. Mice were vaginally infected with C.t., and 5 weeks post challenge, the mice were euthanized. Cells from the GT tissue were stimulated *in vitro* with C.t. peptides, and CD8^+^ T cell responses were measured by flow cytometry. Out of the five immunogenic epitopes, two epitopes, p11 and p13 (GroES_65–73_ and IncA_60–68_), were recognized by CD8^+^ T cells from infected mice (Figures 2D and 2E). This also demonstrated that peptides p1, p14, and p19 (OmpH_89–97_, IncA_74–82_, and ArtJ_170–178_) were C.t. epitopes that did not prime infection-induced CD8^+^ T cells during infection. The status of epitopes elicited by infection or only by CAF09b vaccination was independent of peptide-binding stability to the H2-D^b^ molecule, as epitopes p11 (infection-induced) and p1 (only CAF09b-induced) exhibited similar half-lives (Figure 2F).

Taken together, we successfully identified five novel CD8^+^ T cell epitopes in four different C.t. proteins, of which two were also recognized following C.t. infection.

### Vaccine-induced CD8^+^ T cells show enhanced recruitment following a genital C.t. infection and are cytotoxic and produce cytokines but are unable to protect against infection

**Figure 3:**
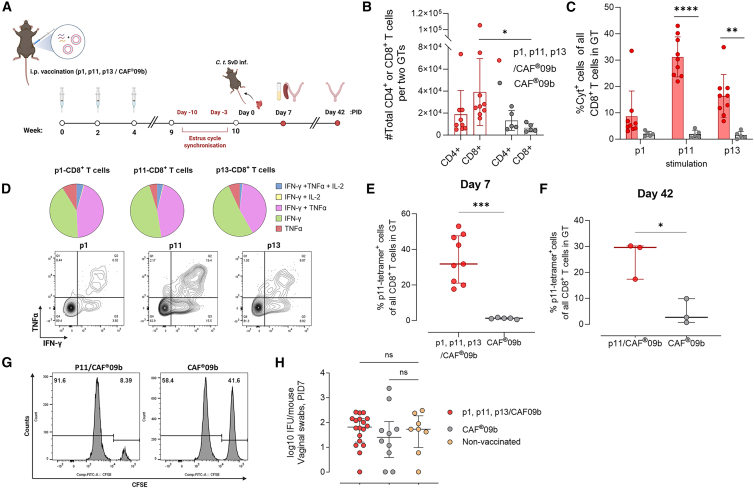
Polyfunctional vaccine-induced CD8^+^ T cells with cytotoxic potential are rapidly recruited to the GT but are unable to afford protection against C.t. infection (A) Study design of the *Chlamydia* challenge experiments (5 × 10^4^ IFUs) evaluating post-infection responses in the murine B6CF31 model. (B) Comparison of absolute T cell numbers in GT tissue (*n* = 10/18 biological replicates, pooled pairwise, mean ± SD, unpaired *t* test) at day 7 post infection measured by flow cytometry. (C) Comparison of antigen-specific CD8^+^ T cell frequencies (*n* = 10/18 biological replicates, pooled pairwise, mean ± SD, unpaired *t* test) identified by Boolean gating analysis of IFN-γ, TNF, and/or IL-2 positivity (% Cyt^+^) at day 7 post infection. (D) Pies showing average proportion of each combination of cytokine expression with representative contour plots over TNF-α and IFN-γ expression in CD8^+^ T cells from the GT stimulated with p1, p11, or p13 at day 7 post infection. (E and F) (E) Comparison of p11-tetramer^+^ CD8^+^ T cells in the GT of peptide/CAF09b- and CAF09b-vaccinated animals at day 7 (*n* = 10/18 biological replicates, pooled pairwise, mean ± SD, unpaired *t* test) and (F) day 42 post infection (*n* = 3 biological replicates, mean ± SD, unpaired *t* test). (G) Representative histograms showing specific lysis of CFSE-labeled splenocytes *in vivo* after 20 h, in which “Target” p11-loaded splenocytes (CFSE^high^) and “Non-target” unloaded splenocytes (CFSE^low^) were transferred into animals vaccinated with p11/CAF09b or CAF09b. (H) Bacterial burden at day 7 post infection was determined through bacterial cultivation of swab samples. GT bacterial numbers are presented as log10 of IFUs (*n* = 8/10/18 biological replicates, median ± IQR, Kruskal-Wallis test with Dunn’s multiple comparison test). PID, post infection day; i.p., intraperitoneal; Cyt^+^, cytokine positive; CFSE, carboxyfluorescein succinimidyl ester; IQR, interquartile range; GT, genital tract; IFUs, inclusion forming units. Statistical significance is represented by ns, non-significant, ∗*p* < 0.05, ∗∗*p* < 0.01, ∗∗∗*p* < 0.001, ∗∗∗∗*p* < 0.0001.

Having identified CD8^+^ T cell-specific responses against C.t., we next investigated the post-infection responses of vaccine-induced CD8^+^ T cells. Here, we initially focused on the three most immunogenic peptides, OmpH_89–97_ (p1) GroES_65–73_ (p11), and IncA_60–68_ (p13) (Figure 2C), of which the latter two are infection-induced epitopes (Figure 2D). Mice were immunized three times with a mix of p1, p11, and p13 formulated in CAF09b or with CAF09b alone as control. Six weeks post final immunization, mice were infected by the vaginal route with 5 × 10^4^ inclusion forming units (IFUs) of C.t. SvD (Figure 3A).

Mice that received the CD8^+^ peptide vaccine (p1, p11, or p13/CAF09b) exhibited increased numbers of CD8^+^ T cells in the GT on day 7 post infection compared to CAF09b control mice (Figure 3B). *In vitro* stimulation with peptide antigens p1, p11, or p13 showed that among the cytokine-expressing CD8^+^ T cells (TNF-α, IFN-γ, and/or IL-2), 8.7%, 31.2%, and 16.4%, respectively, were specific for the peptides (Figure 3C). Consistent with this, stimulation of splenocytes from vaccinated mice also resulted in a significant release of IFN-γ (Figure S3A). As expected, the vaccine did not induce specific antibody responses against any of the epitopes (Figure S3B).

By using an MHC-I tetramer (H2-D^b^) against the infection-induced epitope p11 (GroES_65–73_), we found that the frequency of tetramer-positive cells (Figure 3E) was similar to that of p11 cytokine-positive cells at day 7 post infection (Figure 3C), indicating that all vaccine-induced CD8^+^ T cells were capable of producing cytokines (Figure 3E). Expression analysis revealed that the majority of cytokine-positive CD8^+^ T cells, at day 7 of infection, were IFN-γ^+^ (40%–50%) or IFN-γ^+^/TNF-α^+^ (40%–45%) (Figure 3D). Furthermore, on day 42 post infection, the vaccine-induced CD8^+^ T cells were still present (Figures 3F and S3C). Lastly, at day 30 post infection, approximately 40% of the P11-specific CD44^hi^ CD8^+^ T cells co-expressed Trm markers CD103 and CD69, and 30% and 7% expressed CD69 or CD103 alone, respectively. The remaining 20% lacked expression of Trm markers (Figure S3D), consistent with a non-Trm memory phenotype. Although CAF09b control mice had a lower level of Cyt+ P11-specific CD8^+^ T cells (Figures 3F and S3C), they displayed a similar CD69 and CD103 co-expression pattern as the p11/CAF09b-vaccinated animals (Figure S3E).

In addition to being recruited to the GT and possessing the ability to produce cytokines, we next investigated whether the CAF09b adjuvant also generated CD8^+^ T cells that exhibited cytotoxic capabilities. To examine this, mice were immunized with p11/CAF09b or with CAF09b alone, and 1 week after final immunization, mice were injected intravenously with equal numbers of CFSE^high^-labeled (p11-pulsed) and CFSE^low^-labeled (non-pulsed) splenocytes from naive mice. After 20 h, specific lysis of the transferred cells was determined by flow cytometry analysis from splenocytes of recipient vaccinated mice (Figure 3G). We observed 84.1% ± SD 5.8% specific killing of the p11-loaded target cells (CFSE^high^) compared to non-loaded cells (CFSE^low^) in p11/CAF09b-vaccinated mice (Figure S4C), demonstrating strong cytotoxic ability of CAF09b-induced CD8^+^ T cells.

Having demonstrated the functional abilities of vaccine-induced CD8^+^ T cells and their fast recruitment to the infected GT, we next examined the protective efficacy of the vaccine-induced CD8^+^ T cells. Bacterial quantification of vaginal swabs collected from infected, p1-, p11-, p13/CAF09b-vaccinated, or control mice revealed no significant reduction in the bacterial burden at day 7 post infection in vaccinated mice (Figure 3H). When the infection-induced epitope and the other immunogenic epitope were separated by individual vaccination of p1 and p11 (p1/CAF09b and p11/CAF09b), no reduction in the bacterial level was observed compared to control mice (Figure S3D). Moreover, mice vaccinated with p11/CAF09b exhibited levels of gross GT pathology similar to those observed in non-vaccinated mice or CAF09-vaccinated mice (Figure S6). Finally, supplementing a CD4-inducing vaccine CTH522/CAF01 with a CD8-inducing vaccine (p1 or p11/CAF09b) did not lead to increased protection (Figure S3F).

Taken together, following infection, vaccine-induced CD8^+^ T cells with cytolytic potential demonstrated rapid recruitment to the GT, where they exhibited a polyfunctional cytokine production capacity and persisted following infection. However, the vaccine-induced CD8^+^ T cells did not contribute to early protection against infection and did not affect the development of pathology.

### Vaccination with distinct combinations of CD8^+^ T cell epitopes does not confer protection against a C.t. infection

So far, we have presented five different C.t. CD8^+^ T cell epitopes. The three most immunogenic epitopes were formulated in CAF09b and generated CD8^+^ T cells with multifunctional abilities. Nevertheless, vaccination with these did not reduce bacterial levels following C.t. infection.

Given the significant implications of a non-protective role for CD8^+^ T cells against a C.t. infection, we investigated additional epitope combinations. We divided the peptides into the following three groups: infection-induced epitopes (p11 and p13), less immunogenic epitopes (p14 and p19), and finally a combination of all the immunogenic epitopes. The UV-irradiated C.t. SvD (UV-SvD) or CTH522/CAF01 vaccines, which predominantly induce CD4^+^ T cell responses, were included as positive controls.^13^^,^^57^ In all the experiments, mice were immunized as described earlier followed by vaginal challenge with C.t. SvD 6 weeks post last immunization.

**Figure 4:**
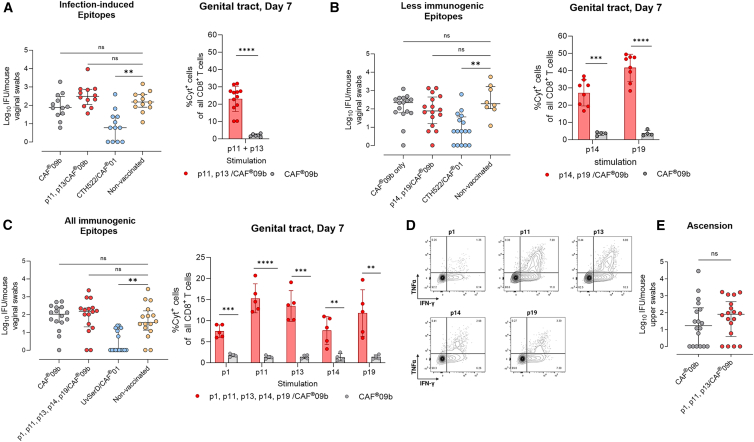
Different vaccine combinations of CD8^+^ T cell epitopes do not provide protection against genital C.t. infection Groups of female B6C3F1 mice were vaccinated as indicated with 10–30 μg per CD8^+^ T cell peptide antigen, 5 μg CTH522, or 5 μg UV-SvD formulated in CAF09b or CAF01. Mice received three vaccine doses at 2-week intervals over a 6-week period. Vaccine was given either intraperitoneally with CAF09b- or subcutaneously with CAF01-formulated vaccines. Six weeks after final immunization, mice were challenged vaginally with 5 × 10^4^ IFUs of C.t. SvD. Bacterial loads were assessed at day 7 post infection, by culturing samples obtained from vaginal swabs. (A–C) Bacterial burden (median ± IQR, Kruskal-Wallis with Dunn’s multiple comparison test) (left) and frequencies of Cyt^+^ CD8^+^ T cells in the GT were measured by flow cytometry (right, mean ± SD, unpaired *t* test). (A) Data points represent individual GT in the p11, p13/CAF0b group (*n* = 12 biological replicates) or pairwise-pooled GT in the CAF09b group (*n* = 12 biological replicates). (B) Data points represents pairwise-pooled GT in the p14, p19/CAF09b group (*n* = 16 biological replicates), or individual GTs in the CAF09b-group (*n* = 4 biological replicates). (C) Data points represent GTs pooled 3, 3, 3, 3, and 4 (*n* = 16 biological replicates) in the p1, p11, p13, p14, p19/CAF09b group and GT pooled in groups of four (*n* = 16 biological replicates) in the CAF09b group. (D) Representative flow analysis contour plots over TNF-α^+^, IFN-γ^+^ CD8^+^ T cells in the GT from experiment shown in (C). (E) Ascension of bacteria. Bacterial burden (median ± IQR, *n* = 16 biological replicates) in the uterine horns at day 7 post infection. CTH522, *C. trachomatis* hybrid 522 antigen; UV-SvD, UV-irradiated *C. trachomatis* serovar D; Cyt^+^, cytokine positive; IFUs, inclusion forming units; IQR, interquartile range; GT, genital tract. Statistical significance is represented by ns, non-significant, ∗∗*p* < 0.01, ∗∗∗*p* < 0.001, ∗∗∗∗*p* < 0.0001.

When evaluating the vaccine efficacy of the two infection-induced CD8^+^ T cell epitopes, p11 and p13, no significant reduction in bacterial levels was observed following infection, in contrast to animals that received the CD4^+^ T cell-inducing vaccine CTH522/CAF01 (Figure 4A). In addition, the combination of less immunogenic epitopes, p14 and p19, did not lead to protection against infection (Figure 4B). Finally, in mice vaccinated with all five immunogenic epitopes, the bacterial levels were similar to those of non-vaccinated mice (Figure 4C). Notably, the absence of protection against C.t. infection was observed despite the presence of significant local CD8^+^ T cell responses (Figures 4A–4D).

We also measured the bacterial load in the upper GT of p1-, p11-, and p13/CAF09b-vaccinated mice to assess whether the CD8^+^ T cell vaccine prevented ascension of the bacteria to the uterine horns. However, CD8^+^ T cell vaccination did not reduce the bacterial burden in the upper GT, indicating a lack of protection against ascending C.t. infection (Figure 4E). Finally, as the inhibitory receptor programmed death-1 (PD-1) and its ligand PD-L1 have been implicated in the impairment of CD8^+^ T cell function during C.t. infection,^58^^,^^59^ we speculated whether antibody-mediated blockage of PD-L1 would enable vaccine-induced CD8^+^ T cells to protect against vaginal infection in mice vaccinated with infection-induced CD8^+^ T cell epitopes (p11 and p13/CAF09b). However, despite a significant expression of PD-L1 in uterine epithelial cells during C.t. infection, blocking PD-L1 did not result in protection against infection (Figure S7).

In conclusion, evaluation of various combinations of immunogenic C.t. CD8^+^ T cell epitopes from different C.t. proteins demonstrated that none of the CD8^+^ T cell vaccines conferred protection against vaginal C.t. infections.

### Inducing CD8^+^ T cells recognizing MOMP

**Figure 5:**
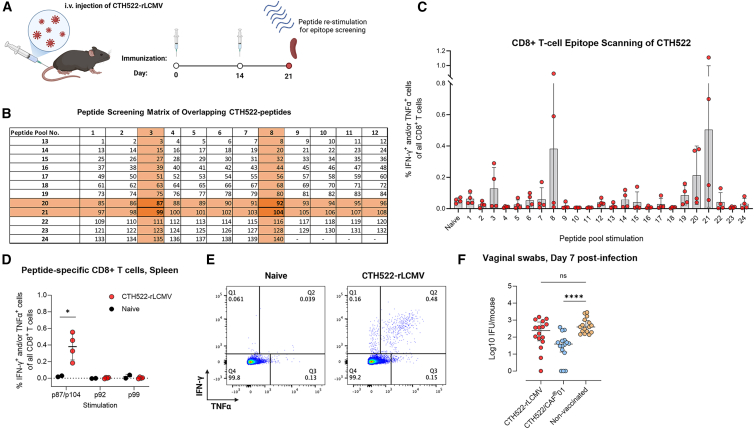
Induction of MOMP-specific CD8^+^ T cells with rLCMV does not contribute protection against C.t. infection (A) Schematic illustration of the CTH522 epitope scanning experiment. C57BL/6J mice were immunized intravenously with 1 × 10^6^ FFU rLCMV-CTH522 on days 0 and 14 (*n* = 8/16 biological replicates, pooled in groups of four). Spleens were harvested 1 week after final immunization and stimulated with pools of overlapping peptides or individual peptides from CTH522. (B) CTH522 peptide screening matrix of 140 overlapping peptides (15-mers with 11aa overlap) spanning the full protein. Twenty-four peptide pools overlapping with one single peptide, where each peptide was included in two different pools, creating a two-dimensional matrix for detection of immunogenic epitopes. (C) Frequencies of IFN-γ^+^ and/or TNF-α^+^ CD8^+^ T cells from spleen cells (mean ± SD) re-stimulated with the peptide pools (final concentration 10^−6^ M/peptide). (D) Total percentages of IFN-γ and/or TNF-α-producing CD8^+^ T cells (mean ± SD) from vaccinated mice upon stimulation with individual peptide p87/104, p92, or p99. (E) Representative dot plots over splenic IFN-γ^+^ and/or TNF-α^+^ CD8^+^ T cells. (F) Day 7 post infection bacterial burden (median ± IQR) of vaccinated and vaginally challenged (5 x 10^4^ IFUs) B6C3F1 mice (*n* = 16 biological replicates). i.v., intravenous; CTH522, *Chlamydia trachomatis* hybrid 522 antigen; rLCMV, recombinant lymphocytic choriomeningitis virus; FFUs, focus forming units; aa, amino acid; IQR, interquartile range. Statistical significance is represented by ns, non-significant, ∗*p* < 0.05, ∗∗∗∗*p* < 0.0001.

The major outer membrane protein (MOMP) is the most abundantly expressed protein on the surface of C.t.^60^ and is recognized for its capacity to stimulate adaptive immune responses during infection.^61^^,^^62^^,^^63^ To identify MOMP CD8^+^ T cell epitopes, we conducted an *in silico* prediction of MOMP from SvD and selected 11 epitopes (9- and 10-mers) (Figure S8A). The selected epitopes were grouped into pools of three and were formulated in CAF09b for vaccination as described earlier. Following immunization, splenocytes were taken out and stimulated with the individual epitopes for detection of cytokine-positive CD8^+^ T cell responses. However, peptide vaccination with CAF09b failed to elicit MOMP-specific responses (Figure S8B). We therefore selected an alternative approach to generate MOMP-specific CD8^+^ T cells. We used the rLCMV, as this vector is known for its ability to generate robust CD8^+^ T cell responses.^64^ The MOMP-based antigen CTH522^13^^,^^54^ was incorporated into the lymphocytic choriomeningitis virus (LCMV) vector. Using a homologous prime-boost regime, C57BL/6J mice were immunized with 1 × 10^6^ focus forming units of CTH522-rLCMV with a 14-day interval. One week following the last boost, splenocytes were isolated for analysis of antigen-specific responses (Figure 5A). To identify CD8^+^ T cell epitopes in CTH522, we conducted epitope scanning using 140 overlapping peptides spanning the CTH522 sequence (15-mers overlapping with 11 amino acids). For the peptide screening, a two-dimensional peptide matrix was created in which all 140 peptides were divided into 24 peptide pools. Each and every peptide was included in two separate pools, in which the pools overlapped by one single peptide. Thus, a positive signal in two individual pools would allow us to identify individual epitopes. From these pool stimulations, peptides p87, p92, p99, and p104 were found in the four pools that produced positive cytokine signals. These peptides were not among the 11 previously predicted epitopes (Figures 5B and 5C). Furthermore, peptides p87 and p104 had identical sequences (as the VD4 region from SvD, E, F, and G in CTH522 shares high homology^13^).

Re-stimulation of splenocytes from CTH522-rLCMV-vaccinated mice revealed that only peptide p87/104 (DTMQIVSLQLNNMFT) elicited significant positive CD8^+^ T cell responses (Figures 5D and 5E). Interestingly, p87 was also recognized in infected mice and thus constituted an infection-induced epitope (Figure S8C). Having identified the induction of MOMP-specific CD8^+^ T cells, we conducted an independent experiment to evaluate the protective efficacy of CTH522-rLCMV. B6C3F1 mice were immunized with CTH522-rLCMV and subsequently challenged with C.t. SvD. On day 7 post infection, we assessed the influx of T cells and bacterial load. Consistent with the results observed using CAF09b, LCMV vaccination resulted in enhanced recruitment of CD8^+^ T cells to the GT following infection (Figure S8D). However, analysis of bacterial counts in vaginal swabs revealed no statistically significant reduction in the bacterial burden of LCMV-vaccinated mice, demonstrating that MOMP-p87-specific CD8^+^ T cells also did not confer protection against infection (Figure 5F).

## Discussion

The conflicting findings regarding the role of CD8^+^ T cells in combating *Chlamydia* infection^27^^,^^28^^,^^29^^,^^31^^,^^32^ warranted a comprehensive investigation of vaccine-elicited CD8^+^ T cells to determine their protective potential. Following C.t. infection, we showed that CD8^+^ T cells are induced by infection and recruited to the GT, with delayed kinetics compared to CD4^+^ T cells, and persisted in the GT (Figure 1A). Moreover, IHC analysis further demonstrated that these late-stage CD8^+^ T cells were in close contact with the uterine epithelium (Figures 1B and 1C), which is similar to the location of intraepithelial CD8^+^ T cells in other tissues such as the gut mucosa.^65^ This accumulation of CD8^+^ T cells shows that C.t. can induce CD8^+^ T cell responses during primary infection, as previously observed in both humans and other experimental animal infection models.^66^^,^^67^^,^^68^^,^^69^

A significant challenge in investigating antigen-specific responses is to discover immunogenic epitopes, particularly those induced by infection. A limited number of immunodominant CD8^+^ T cell epitopes have previously been reported in murine C.t. infections, namely Cap1, CrpA, and PmpI.^24^^,^^34^^,^^35^ Cap1_139–147_ and CrpA_63–71_ were identified from serovar L through a retroviral expression-cloning strategy,^24^^,^^34^ whereas PmpI_612–620_ was discovered during infection with serovars L and D via the use of caged MHC class I molecules.^35^ We also predicted an epitope in CrpA, in the same region, but were not able to generate CD8^+^ T cells against it with the liposomal CAF09b adjuvant (Figure 2B).

Here, we identified three novel immunodominant epitopes p11, p13, and p87 recognized in B6CF1 mice (H2-D/H2-K^b,k^) (Figures 2D, 2E, and S7C). The first two identified epitopes (GroES_65–73_ and IncA_60–68_) are part of the heat shock protein CT111 and the inclusion membrane protein CT119, respectively, which have been shown to be among the most abundantly expressed C.t. proteins.^70^ Peptide p87, discovered in the peptide scanning of CTH522, is found in the variable domain 4 of MOMP.^71^ In addition, we also identified three other immunogenic epitopes, OmpH_89–97_, IncA_74–82_, and ArtJ_170–178_ (p1, p14, and p19) (Figure 2C), which were not recognized by CD8^+^ T cells during infection (Figure 2D). The induction of these epitopes was possible by using the liposomal CAF09b adjuvant that is able to activate dendritic cells (DCs) and induce cross-presentation of antigens.^36^ Inclusion of cryptic T cell epitopes in vaccine strategies has shown to participate in protection against intracellular infections by broadening the CD8^+^ T cell repertoire,^72^ or in terms of CD4^+^ T cell epitopes, by improving CD4^+^ T cell responses to subdominant epitopes.^73^^,^^74^ However, whether these epitopes are indeed cryptic remains to be shown.

Our data showed that immunization with the epitopes OmpH_89–97_, GroES_65–73_, and IncA_60–68_ (p1, p11, and p13) when formulated in CAF09b elicited CD8^+^ T cells that, following vaginal challenge, were recruited to the GT and exhibited an ability to persist there (Figures 3B–3E). Once in the GT, the cells displayed multifunctional capacity, particularly in terms of cytokine production of IFN-γ and TNF-α (Figure 3D). In agreement with our findings, a previous study demonstrated that antigen-specific CD8^+^ T cells during murine genital C.t. infection migrated to the infection site, where they possessed the ability to produce IFN-γ.^75^ Furthermore, immunizing NHPs with a viral vector encoding CTH522 also led to the induction of CD8^+^ T cells capable of producing IFN-γ alone, or IFN-γ and TNF-α.^31^

Using the liposomal adjuvant CAF09b, we induced CD8^+^ T cells with strong *in vivo* cytolytic potential (Figure 3G). Correspondingly, early *in vitro* studies isolating CD8^+^ T cells from convalescent individuals or C.t.-infected mice demonstrated an ability of CD8^+^ T cells to kill C.t.-infected and C.t.-peptide pulsed cells, respectively.^24^^,^^34^^,^^76^ However, whether these vaccine-induced CD8^+^ T cells are capable of exerting their cytotoxic function in the context of an infected genital mucosa through recognition of their epitope remains to be shown.

Vaccination with the three most immunogenic epitopes (OmpH_89–97_, GroES_65–73_, and IncA_60–68_) did not elicit protective CD8^+^ T cell responses against genital C.t. infection (Figure 3H). Furthermore, vaccination with epitopes based on their immunodominance, either individually or in combination, failed to provide significant protection against infection, despite the presence of local antigen-specific responses (Figures 4A–4C). Additionally, none of the CD8^+^ T cell vaccines protected against ascension to the upper GT (Figure 4E). Finally, immunization with the viral-based vector rLCMV expressing the MOMP-based CTH522 antigen led to MOMP-specific CD8^+^ T cells but no protection (Figures 5D and 5F).

Although we cannot exclude that CD8^+^ T cells specific for other antigens may contribute to protection, our observations do align with a recent study that investigated various replication-deficient viral vectors incorporating C.t. antigens CPAF and OmcB.^77^^,^^78^ The study found that CD8^+^ T cell-dominant responses induced by these vectors either failed to induce protection against infection with *Chlamydia muridarum* or only contributed to a marginal reduction in bacterial burden of infected mice.^77^ In contrast, vaccine-induced CD8^+^ T cells did exhibit a protective role against ocular infection in NHPs immunized with a live-attenuated C.t. vaccine.^32^ While ocular and genital chlamydial infections share common protective immune mechanisms, such as induction Th1 responses and IFN-γ production, they also exhibit tissue-specific differences in conjunctival and genital mucosa, which influence the local immune environment.^79^ Moreover, in contrast to mice, NHPs are known to exhibit more CD8^+^ T cell-orientated immune responses.^80^

In our model, it could be speculated that the lack of CD8^+^ T cell-mediated protection is due to an inhibition of these cells in the infected tissue. A study on genital C.t. infection demonstrated that CD8^+^ T cell responses were inhibited by the upregulation of PD-L1, in which CD8^+^ T cell-dependent clearance of C.t. was observed in PD-L1-deficient animals. Furthermore, CD8^+^ T cells in these PD-L1-deficient mice exhibited enhanced IFN-γ production and improved recall responses, highlighting the negative effect of PD-L1 expression on CD8^+^ T cell function.^58^ However, blocking the PD-1/PD-L1 interaction had no effect in our model (Figure S7). The secretion of anti-inflammatory IL-10 has been shown to be influenced by *Chlamydia* infection and appears to play a significant role in its pathogenesis.^81^ *In vitro* studies have previously revealed that *Chlamydia*-induced IL-10 secretion led to the downregulation of MHC class I but not MHC class II expression, suggesting a potential mechanism for the inhibition of CD8^+^ T cell-mediated protection.^82^ We did however not see any significant downregulation of MHC-I (data not shown).

In conclusion, our investigation of post-infection responses to multiple CD8^+^ T cell epitopes revealed that, although CD8^+^ T cells were effectively recruited to the GT and exhibited functional effector mechanisms, they did not confer protection against genital C.t. infection. In contrast, protective immunity was associated with CD4^+^ T cell responses induced by vaccination. These findings underscore the importance of prioritizing CD4^+^ T cell-mediated immunity and antibody responses in the design of effective *Chlamydia* vaccines and suggest a limited protective role for CD8^+^ T cells.^71^^,^^77^

### Limitations of the study

Testing five CD8^+^ T cell epitopes that all proved non-protective does not imply that all CD8^+^ T cell epitopes will be non-protective. To reach such a conclusion, a larger number of CD8^+^ T cell epitopes would need to be tested. Furthermore, whether the C.t. CD8^+^ T cell epitopes are efficiently processed and presented on MHC-I molecules on infected epithelial cells, and whether they are identical to those presented by DCs via cross-priming, were not investigated in this study. This constitutes a limitation of the current study.

## Resource availability

### Lead contact

Further information and requests for resources and reagents should be directed to and will be fulfilled by the lead contact, Nina Dieu Nhien Tran Nguyen (ndtn@ssi.dk).

### Materials availability

The study did not generate new unique reagents. All other data are available upon request from the lead contact.

### Data and code availability

• All data reported in this paper will be shared by the lead contact upon request. This includes flow cytometry FCS files, IFU quantification data, ELISA absorbance values, MHC I-peptide stability assay data, and cytotoxicity assay data.
• This paper does not report original code.
• Any additional information required to reanalyze the data reported in this paper is available from the lead contact upon request.

## Acknowledgments

This study was primarily supported by the European Union’s 10.13039/501100007601Horizon 2020 research and innovation program under the Marie Sklodowska-Curie grant agreement no. 812915 and received additional support from 10.13039/501100005747A.P. Møller Fonden: Fonden til Lægevidenskabens Fremme (grant no. L-2021-00156). The funders played no role in study design, data collection, analysis and interpretation of data, or the writing of this manuscript. The authors express their sincere gratitude to Lene Rasmussen and Anne Bjørlig for providing technical support and to Emma Lorenzen for scoring the cell influx on the immunohistochemical slides. Additionally, they extend their appreciation to Annika de Caprétz and the entire team of animal technicians at the Statens Serum Institut for their valuable contributions.

## Author contributions

J.D., F.F., and S.G. conceived and designed the experiments. S.G., N.D.N.T.N., S.S., M.C., A.L.K., E.N., and K.M.M. performed the experiments. S.G., J.D., D.P., J.P.C., A.S., N.D.N.T.N., F.F., A.W.O., and K.M.M. analyzed the data. S.G., J.D., F.F., and N.D.N.T.N. drafted and edited the paper. Each of the listed co-authors made substantial contributions to the work through design and conception and/or acquisition, analysis, and interpretation of the data. All authors reviewed the manuscript.

## Declaration of interests

A.W.O. and F.F. are co-inventors of a patent (WO2014146663A1) related to C.t. vaccines. All rights have been assigned to Statens Serum Institut, a Danish not-for-profit governmental institute.

## STAR★Methods

### Key resources table

**Table:** 

REAGENT or RESOURCE	SOURCE	IDENTIFIER
**Antibodies**		

Anti-mouse H-2Db-biotin (clone 28-14-8)	Invitrogen	Cat# 13-5999-82; RRID: AB_466875
rabbit anti-MOMP SvD	In-house	N/A
Anti-rabbit IgG-Alexa Flour 488 polyclonal	Life Technologies	Cat# A-11008; RRID: AB_143165
Anti-mouse CD28 (clone 37.51)	BD Biosciences	Cat# 553294; RRID: AB_394763
Anti-Mouse CD16/CD32 (clone 2.4G2)	BD Biosciences	Cat# 553141; RRID: AB_394656
Anti-mouse CD8a-BV421 (clone 52–6.7)	BD Biosciences	Cat# 563898; RRID: AB_2738474
Anti-mouse CD8a-PerCP-Cy5.5 (clone 52–6.7)	Invitrogen	Cat# 45-0081-82; RRID: AB_1107004
Anti-mouse CD4-BV786 (clone GK1.5)	BD Biosciences	Cat# 563331; RRID: AB_2738140
Anti-mouse CD44-APC (IM7)	BD Biosciences	Cat# 559250; RRID: AB_398661
Anti-mouse CD44-Alexa Fluor 700 (IM7)	Biolegend	Cat# 103026; RRID: 103026
Anti-mouse CD69-PE-Cy7 (clone H1.2F3)	BD Biosciences	Cat# 561930; RRID: AB_394508
Anti-mouse CD103-PE (Clone M290)	BD Biosciences	Cat# 561043; RRID: AB_396732
Anti-mouse CD49a-BV421 (Ha31/8)	BD Biosciences	Cat# 740046; RRID: AB_2739815
Anti-mouse CD107a-BV786 (1D4B)	BD Biosciences	Cat# 564349; RRID: AB_2738762
Anti-mouse CD45.2-FITC (Clone 104)	BD Biosciences	Cat# 553772; RRID: AB_395041
Anti-mouse CD3e BV650 (500A2)	BD Biosciences	Cat# 740461; RRID: AB_2740187
Anti-mouse IFNγ-PE-Cy7 (clone XMG1.2)	BD Biosciences	Cat#: 557649; RRID: AB_396766
Anti-mouse IFNγ-PE-CF594 (clone XMG1.2)	BD Biosciences	Cat#: 562303; RRID: AB_11153140
Anti-mouse IL-2-APC-Cy7 (clone JES6-5H4)	BD Biosciences	Cat# 560547; RRID: AB_1727544
Anti-mouse TNFα -APC (clone MP6-XT22)	BD Biosciences	Cat# 554420; RRID: AB_398553
Rabbit Anti-Mouse IgG(H + L)-HRP	AH Diagnostics	Cat# 6170-05;
Rat Anti-mouse IFNγ (clone R4-6A2)	BD Biosciences	Cat# 551216; RRID: AB_394094
Anti-mouse IFNγ-Biotin (clone XMG1.2)	BD Biosciences	Cat# 554410; RRID: AB_395374
Rabbit Anti-mouse CD4 (clone R001)	Nordic Biosite	Cat# 50134-R001; RRID: AB_2860490
Rabbit Anti-mouse CD8 (clone poly)	Nordic Biosite	Cat# bs-0648R; RRID: AB_10857537

**Bacterial and virus strains**		

*Chlamydia trachomatis* (SvD/UW-3/Cx)	ATCC	VR-885
rLCMV-CTH522	This study	N/A

**Chemicals, peptides, and recombinant proteins**		

Chlamydia trachomatis hybrid (CTH)522	In-house^83^	N/A
Peptide 1-20	JPT peptides Technologies	N/A
CTH522 15-mer peptides 11 aa overlap	Genscript Biotech	N/A
Viakrome 808 Fixable Viability Dye	Beckman Coulter	Cat# C36628
Streptavidin-PE	eBioscience	Cat# 12-4317-87
Propidium Iodide	Sigma-Aldrich	Cat# P4170
Fixable Viability Dye eFluor™ 506	eBioscience	Cat# 65-0866-18
MHC I Tetramer H2-K^b^p11-APC	Donated by Jan P. Christensen	N/A
5(6)-Carboxyfluorescein diacetate N-succinimidyl ester CFSE	Sigma-Aldrich	Cat# 21888
Streptavidin-HRP	BD Biosciences	Cat# 550946; RRID: AB_2868972

**Critical commercial assays**		

Cytofix/Cytoperm Solution Kit	BD Biosciences	Cat# 554714

**Experimental models: Cell lines**		

HeLa-229 CCL-2.1™	ATCC	RRID:CVCL_1276
McCoy cells CRL-1696™	ATCC	RRID:CVCL_3742
GP-expressing BHK21 cells	In-house^37^	N/A
RMA-S cells	In-house^84^	RRID:CVCL_2180
GP-expressing HEK293T cells	In-house^37^	N/A

**Experimental models: Organisms/strains**		

Mouse: B6C3F1/OlaHsd (H-2b,k)(C57BL/6JOlaHsd inbred female x C3H/HeNHsd inbred male)	Envigo (now Inotiv)	946
Mouse: C56Bl/6J (JAX:000664)	Charles River	027

**Software and algorithms**		

FlowJo v10	BD Biosciences	www.flowjo.com
Graphpad Prism v10	Graphpad Software	www.graphpad.com
NetMHCpan-4.1	DTU Health Tech	www.services.healthtech.dtu.dk/services/NetMHCpan-4.1/
CellReporterXpress®	Molecular Devices	Molecular Devices
BioRender	Biorender	Follmann, F. (2026) https://BioRender.com/7j3l8ks
Caseviewer version 2.3	3Dhistech	3Dhistech

**Other**		

CAF®01	In-house^85^	N/A
CAF®09b	In-house^86^	N/A

### Experimental model details

#### Ethics statements

Experiments conducted at Staten Serum Institut were performed in compliance with regulations established by the Danish Ministry of Justice and animal protection committees, as authorized by Danish Animal Experiments Inspectorate Permit 2020-15-0201-00637. The studies adhered to the European Community Directive 2010/63/EU of the European Parliament and Directive 86/609, as well as the recommendations set forth by the U.S. Association for Laboratory Animal Care regarding the use of laboratory animals. The experiments were approved by a local animal protection committee at Statens Serum Institut, IACUC, headed by DVM Kristin Engelhart Illigen. All methods are reported in accordance with ARRIVE guidelines. Mice experiments that took place in University of Basel were performed in accordance with the Swiss law for animal protection and with permission by the Cantonal Veterinary Office of Basel City.

#### Animals

Animal studies were performed on 6- to 8-week-old female B6C3F1 hybrid mice (H-2D/H-2K^b, k^) obtained from Envigo, Scandinavia (strain #946). Upon arrival, mice were assigned cages in a random matter and acclimatized for at least one week. All mice were fed radiation sterilized 2016 Global Rodent Maintenance diet (Envigo, 2916C) and water *ad libitum*. Animals were housed at an ambient temperature of 20°C–23 °C and 45–65% relative humidity on a 12 h/12 h light/dark cycle with 15 min dusk and dawn transition periods under Biosafety Level (BSL) II and handled by authorized full-time personnel at Statens Serum Institut. Mice had access to nesting material (enviro-dri and soft paper wool; Brogaarden) as well as enrichment (aspen bricks, paper house, corn, seeds, and nuts; Brogaarden). Animals were handled by non-aversive handling (such as cup- and tunnel-handling) whenever possible. Only female mice were included in the infection studies, as the infection model requires vaginal/transcervical inoculation and assessment of genital tract infection, which cannot be replicated in male mice. Consequently, sex-based comparisons were not performed. Findings should therefore be interpreted within the context of female-specific immunity and disease.

C57BL/6J mice (JAX:000664; Charles River, strain #027) were maintained under specific-pathogen-free (SPF) conditions during maintenance and experiments at the University of Basel. Animals were fed irradiated maintenance chow (M/R Haltung Extrudat rund, >25 kGy, 15 mm pellets; Granovit AG, Switzerland; product code 343900EXF12), provided water *ad libitum* and had access to nesting and enrichment material (bedding, bricks and cellulose shelter, Labodia Enrichment). Mice were housed on a 12 h/12 h light/dark cycle at a room temperature of approximately 22°C–24 °C and relative humidity of approximately 50%, in accordance with Swiss federal regulations and with permission from the Cantonal Veterinary Office of Basel-Stadt. Mice were 10–14 weeks of age at the start of experiments and sex-matched. Animals were not randomized to experimental groups.

### Method details

#### *In silico* predictions of CD8^+^ T cell epitopes

A total of 10 *C. t.* protein candidates recognized as immunodominant in sera and blood of convalescent individuals, or in the setting of experimental chlamydia infection models were selected for *in silico* prediction (Table 1). The CD8^+^ T cell epitope predictions were performed using the online prediction tool NetMHCpan-4.1(https://services.healthtech.dtu.dk/services/NetMHCpan-4.1/) in which twenty 9-mer peptides restricted to MHC class I alleles H-2D^b^, H-2K^b^ or H-2K^k^ were chosen. The prediction outputs were primarily filtered based on %EL_rank (natural eluted ligands) and selected in conjunction with %BA_rank (binding affinity), in which the ranking represents the predicted binding score of the given peptide compared to a set of random natural peptides. All selected 20 peptides were predicted to be strong binders with %EL_Rank <0.5, exhibiting the highest binding level.

#### Antigens, peptides, CAF01 and CAF09b immunization

Selected peptide antigens harboring the predicted CD8^+^ T cell epitopes from *C. t.* proteins was purchased from JPT peptides Technologies (Berlin, Germany). The customized peptides were designed as 9-mers with a purity of >90%. The recombinant MOMP-based CTH522 vaccine antigen,^13^ was produced at Statens Serum Institut in accordance to good manufacturing practice. Overlapping peptides spanning the entire sequence of CTH522 as 15-mer peptides with 11 amino acid overlap was pursed from GenScript Biotech (New Jersey, USA) (Table S2).

Peptides (10 μg, 30 μg or 40 μg/peptide/dose) were adjuvanted with cationic CAF09b liposomes (DDA/MMG/poly(I:C) in 250 μg/50 μg/12.5 μg per dose) in a total volume of 100 μL.^86^ Mice were immunized intraperitoneally, three times at 2-week intervals. To alleviate pain associated with intraperitoneal (i.p.) vaccination, mice were pre-treated with a subcutaneous injection of buprenorphine (0.07 mg/kg, Temgesic) an hour prior to vaccination.

For vaccination with CAF01 (DDA/TDB in 250 μg/50 μg per dose), mice were immunized three times at 2-week intervals but by the subcutaneous route at the base of the tail in a volume of 200 μL with 5 μg CTH522 vaccine antigen. The vaccines were prepared by admixing the peptide or protein antigens with the desired CAF-adjuvant over 30 min with intermittent vortexing to allow electrostatic adsorption of the antigen onto CAF-liposomes. Lastly, mice referred to as non-vaccinated received no treatment.

#### Viral vector preparation and immunization

The replication-deficient rLCMV-CTH522 vector was generated by reverse genetic engineering using a polymerase I-/polymerase II-based plasmid system, which has been described previously.^87^ The cDNA of the viral glycoprotein (GP) was replaced with the CTH522 antigen, using a similar cloning strategy as previously described.^88^ The rLCMV-CTH522 vector was propagated on BHK-21 cells (ECACC) expressing the viral GP^37^ and titrated by immunofocus assay^89^ on HEK293T cells (ECACC) complementing the viral GP.^37^ BHK-21 cells cultured in Dulbecco’s Modified Eagle Medium (DMEM, Pan-Biotech) supplemented with 10% Fetal Bovine Serum (FBS, Pan-Biotech), 10 mM HEPES (Gibco, 15630056), 1 mM Na-Pyruvate (Gibco, 11360070), 0.3 g/L tryptose phosphate broth (Sigma, T8782) at 37°C with 5% CO_2_. HEK293T cells were cultured in DMEM supplemented with 10% FBS and 1x Penicillin-Streptomycin (P/S, Pan-Biotech) at 37°C with 5% CO_2_. The rLCMV-CTH522 vector was administered intravenously to C57BL/6J mice (JAX:000664; Charles River, strain #027) at a dose of 1 x 10^6^ focus forming units (FFU) per mouse and re-administered after 14 days to boost responses. Given the cell lines renowned origin, no additional cell line authentication was performed by the authors. Cell cultures were screened for mycoplasma contamination by PCR on a regular basis and were confirmed to be mycoplasma-free.

#### MHC I – peptide stability assay

RMA-S cells^84^ were cultured in complete RPMI 1640 GlutaMAX (Thermo Fisher Scientific) media supplemented with 5% heat-inactivated fetal bovine serum (FBS) (VWR) and 1% penicillin-streptomycin (Gibco). To measure the stability of the H2-Db-peptide complex, RMA-S cells were cultured overnight with synthetic peptides (1 μM) under serum-free conditions in OptiMEM (Thermo Fisher Scientific) at 37°C at 5% CO_2_. The following day, cells were harvested, washed 3 times in 1x PBS to remove unbound peptides and then cultured in the absence of peptides. The surface expression of H2-Db was measured over time (0 h, 0.5 h, 1 h, 2 h and 4 h) by flow cytometry (CytoFLEX, Beckman Coulter), upon staining with ViaKrome 808 Fixable Viability Dye (Beckman Coulter, 1:2000) and mouse biotin α -H2-Db (BD biosciences, 28-14-8, 1:100), followed by Streptavidin PE-conjugated secondary antibody (eBioscience, 12-4317-87, 1:200), both for 30 min at 4°C. The stability of the complex MHC-epitope is expressed by its half-life (t½) at the cell surface and was graphically estimated from a fitted linear curve based on the mean fluorescence levels of the peptide-pulsed cells. Samples were analyzed on Flowjo software (version 10.9.0). The cell line was confirmed mycoplasma-free prior to culturing. The authenticity of the cell line was confirmed by the lack of TAP peptide transporter expression.

#### Cultivation of bacteria and *Chlamydia* challenge

*C. t.* SvD bacteria (SvD/UW-3/Cx, ATCC) were grown on confluent monolayer of HeLa-229 cells (ATCC) cultured in RPMI 1640 media (Invitrogen) supplemented with 1%HEPES, 1% of Non-essential amino acids (NEAA) (MP Biomedicals), 1% L-Glutamin (Gibco) and 1% sodium pyruvate (Gibco). *C.t.* bacteria were further propagated in the cells for 2–3 days at 37°C with 5% CO_2,_ in which the cells were later harvested and purified of *C.t.* elementary bodies (EBs) as previously referred.^90^ Purified *C.t.* were resuspended in SPG buffer (250 mM Sucrose, 10 mM Na_2_HPO_4_, 5 mM L-glutamic acid) and divided into aliquots at a concentration of 1.4 x 10^7^ IFUs/μL. Aliquots were stored at −80°C until use. HeLa cells were authenticated by short tandem repeat (STR) profiling by the supplier. The cell lines were tested mycoplasma-negative prior to culturing. Infection batch were tested negative for mycoplasma and *C. t*. serotype was confirmed by chromosomal DNA extraction, PCR amplification and sequencing of the *OmpA* gene.

Prior to infection, all mice were subcutaneously treated at day −10 and −3 with 2.5 mg of Medroxyprogesteron acetate (Depo-Provera, Pfizer) for synchronization of the murine estrous cycle and increase mice susceptibility to chlamydial infection. For vaginal challenge experiments, a total of 5 x 10^4^ IFU/mice in 10 μL SPG buffer was deposited in the vaginal vault of the mouse. Transcervical infection was achieved by bypassing the cervix, with a thin, exible probe i.e., nonsurgical embryo transfer (NSET) device (Paratechs), injecting 1 x 10^3^ bacteria directly into the uterine horn lumen.

#### Bacterial burden

Quantification of bacterial burden post *C. t.* challenge have previously been reported in.^71^^,^^91^ In brief, to assess the bacterial load of infected mice, swab samples from the lower and upper genital tract (one swab stick for each uterine horn) were collected from each individual mouse during the course of infection. The swab sticks were stored at −80°C in 600 mL SPG buffer (250 mM Sucrose, 10 mM Na_2_HPO_4_, 5 mM L-glutamic acid) with glass beads. Prior to cultivation, swab samples were vortexed for 2 min to release bacteria from the swab sticks. For cultivation of bacteria in the swab samples, 0.8 x 10^5^ McCoy cells (ATCC) in culture media (RPMI 1640 (Invitrogen), 1% L-glutamine, 1% non-essential amino acids, 1% sodium pyruvate, 70 μM 2-mercaptoethanol, 0.01% Gentamicin (Gibco), 1% HEPES (Gibco), and 5% heat inactivated FBS (VWR)), were seeded in a 48-well plate (Costar) and incubated at 37°C with 5% CO_2_ overnight. Upon 85–90% confluency, cell media was aspirated and replaced with 0.2 mL glucose infection medium (culture media +0.05% glucose), and further incubated at 37°C with 5% CO_2_. Next, cells were infected by adding undiluted and 1:2 diluted swab samples to the wells and centrifuged down at 700 x g for 1 h with no brake at room temperature. The plates were afterward placed at 37°C with 5% CO_2_ for 2 h incubation. Following, the supernatants were aspirated from the wells and replaced with 0.5 mL infection medium with 1:1000 Cycloheximide (Sigma), and cells incubated for 24 h at 37°C. The following day the cells were fixated with 0.4 mL 96% ethanol per well and kept in 0.4 mL 1x PBS overnight at 4°C. The nuclei of the cells were stained with 0.2 mL/well propidium iodide (Sigma) (solution 1:20) that was followed by labeling of the inclusion bodies with 0.25 mL/well of diluted and sterile-filtrated rabbit anti-MOMP Serovar D antibody (in house) for 1 h at room temperature. Lastly, the cells were incubated at room temperature for 1 h with 0.1mL/well Alexa-Flour 488 conjugated secondary antibody goat anti-rabbit IgG (Life Technologies) diluted 1:1000 in 1x PBS 0.1% BSA. IFUs were quantified using ImageExpress PICO (Molecular Devices) and the CellReporterXpress software or counted manually by fluorescence microscopy. McCoy cell lines were confirmed to be mycoplasma-free prior to culturing and kept in accordance with supplier’s specifications. No further authentication was done by the authors.

#### Sample collection and single-cell preparation

To minimize suffering and distress, mice were anesthetized prior to euthanasia with 6.9 μL/g of a Zoletil-mix (2.4 mg/mL Zolazepam, 2.4 mg/mL Tiletamin, 3.8 mg/mL Xylazin, 0.095 mg/mL Buturphanol). 3 min before euthanasia, 250 μL of anti-CD45.2 – fluorescein isothiocyanate (BD Pharmingen, clone 104, 1:100 dil.) were iv. injected into the tail of the mice to label vascular leukocytes. For euthanasia, mice were exposed to CO_2_ (3 L/min for 5–10 min).

Genital tracts, spleen, iliac lymph nodes, and serum were collected from cohorts of 8–16 mice (individually or pooled in groups of 2 or 4 per organ) in RPMI 1640 (Gibco Invitrogen). The genital tracts underwent enzymatic digestion for 45 min at 37°C in 5% CO_2_ using type IV collagenase (0.8 mg/mL) (Sigma) and DNase I (0.08 mg/mL) (Roche) in supplemented RPMI-1640 (50 μM 2-mercaptoethanol, 1% glutamine, 1% sodium pyruvate, 1% penicillin-streptomycin, 1% HEPES, and 10% heat-inactived FBS). For enhanced tissue homogenization, genital tracts were processed using a gentleMACS Dissociator (Miltenyi Biotec) twice before and one time after enzymatic treatment. Single-cell homogenates were prepared by passing organs through a 100 μm nylon filter (Falcon). The resulting cell suspensions were centrifuged (700 x g, 5 min) and washed twice in RPMI 1640 (Gibco Invitrogen). For peripheral blood mononuclear cell (PBMC) isolation, blood samples were collected in EDTA-coated tubes and diluted 1:1 with 1x PBS. The diluted blood was carefully layered onto Lympholyte solution and centrifuged at 800 x g for 20 min at room temperature. The PBMC layer was then harvested and washed twice in RPMI 1640 (Gibco Invitrogen). Cell pellets from all organs were resuspended in supplemented RPMI-1640 containing 50 μM 2-mercaptoethanol, 1% L-glutamine, 1% sodium pyruvate, 1% penicillin-streptomycin, 1% HEPES, and 10% heat-inactivated FBS. Serum was isolated from blood by centrifugation at 10,000 x g for 10 min. Finally, cell counts were performed with automatic Nucleocounter (Chemotec).

#### Antigen stimulation and flow cytometry

For intracellular cytokine analysis, cells were stimulated *ex vivo* for 1 h with CD8^+^ T cell peptide antigen (10 μg/mL) or CTH522 antigen (5 μg/mL) in the presence of 1 μg/mL co-stimulatory antibodies αCD28 (BD Pharmingen, clone: 37.51) and αCD49d (BD Pharmingen, clone: 9C10 (MFR4.B)) at 37 °C, 5% CO_2_. Brefeldin A was added to the cells afterward at a concentration of 10 μg/mL and were subsequently incubated at 37°C for 6 h. For epitope screening of CTH522, splenocytes was stimulated with peptide pools or individual peptides at a final concentration of 10^−6^ M for a total of 5 h at 37 °C, in which after 1 h of incubation, Brefeldin A was added (10 μg/mL) to the stimulated cells. Stimulation plates were kept at 4°C until staining the following day.

Following stimulation cell suspension were then Fc-blocked with anti-CD16/CD32 (Mouse BD Fc Block, 2.4G2, BD Bioscience, 1:100) and subsequently stained with Fixable Viability- Dye eFluor506 (Invitrogen, 1:500) at 4°C for 20 min. Cells were then stained for surface markers diluted in 50% brilliant stain buffer (BD Horizon) 4°C for 25 min with combinations of the following anti-mouse antibodies conjugated to fluorochromes (company, clone, dilution): α-CD8a-BV421 (BD Horizon, 53–6.7, 1:500), α-CD8a-PerCP-Cy5.5 (eBioscience, 53–6.7,1:600), α-CD4-BV786 (BD Biosciences, GK 1.5, 1:600), α-CD44-APC (BD Biosciences, IM7 1:200), α-CD44-Alexa flour 700 (Biolegend, IM7, 1:150), α-CD69-PE-Cy7 (BD Pharmingen, H1.2F3, 1:200), α-CD103-PE (BD Biosciences, M-290, 1:200), α-CD49a-BV421(BD Biosciences, TS2/7,1:200), α-CD107a-BV786 (BD Biosciences, 1D4B, 1:100), α-CD45.3-FITC (BD Biosciences, 104, 1:100).) The gating strategy for the T cell populations are shown in Figure S5A as well as the gating of Trm markers (CD103, CD49a and CD69, Figure S5C).

For tetramer staining, single-cell suspensions were stained with class I MHC tetramer (H2-K^b^p11-APC conjugated, kindly donated by Jan P. Christensen’s laboratory) diluted 1:5 in FACS buffer (1x PBS + 1%FBS) containing Fc-block anti-CD16/CD32 antibody (Mouse BD Fc Block, 2.4G2, BD Bioscience, 1:100) for 25 min at 4 °C. Tetramer staining was followed by surface staining as described above.

Following surface stain, the cells underwent fixation and permeabilization using the Cytofix/Cytoperm Solution Kit (BD Biosciences) according to the manufacturer’s instructions for detection of intracellular proteins. Samples were followed by intracellular staining for α-CD3e-BV650 (BD Biosciences, 17A2, 1:200), α-IFN γ-PECy7 (Thermo Fisher Scientific, XMG1.2, 1:200), α- IFNγ-PE-CF594 (BD Biosciences, XMG1.2, 1:200) α-IL-2-APC-Cy7 (BD Pharmingen, JES6-5H4, 1:200), α-TNFα -APC (BD Pharmingen, MP6-XT22, 1:200). The cytokine gatings are shown in Figure S5B.

Samples were analyzed by Flow cytometry (BD LSRFortessa, BD Bioscience) and FlowJo Software (version 10.9.0). To analyze the cells in the organs we excluded doublets on forward scatter height (FSC-H) and forward scatter area (FSC-A) plot, excluded cell debris on side scatter height (SSC-H) and FSC-H and lastly excluded dead cells using the viability marker.

#### *In vivo* cytotoxicity assay

To assess specific cytotoxicity, B6C3F1 mice were immunized as previously described with p11 peptide (40 μg) adjuvanted in CAF09b or with CAF09b alone. Single splenocyte suspensions from naive donor mice were obtained by passing spleens through 100-μm cell strainers (Falcon) and were washed in RPMI 1640 (Gibco Invitrogen) in accordance with previous sample preparations. Cells were adjusted to 10 x10^6^ cells/ml in RPMI 1640 (Gibco Invitrogen) supplemented with 1% L-glutamine, 1% HEPES, 10% heat-inactived FBS for peptide pulsing of “Target” cells. Next, cells were pulsed with p11 peptide at a final concentration of 10 μg/mL for 1.5 h at 37 °C, 5% CO2, along with un-pulsed cells now known as “Non-target”. Prior to staining with 5(6)-Carboxyfluorescein diacetate N-succinimidyl ester (CFSE) (Sigma-Aldrich), cells were resuspended in 1xPBS and stained in equal volume CFSE^high^ (5 μM) or CFSE^low^ (0.5 μM) dye solution in darkness for 10 min at 37 °C, 5% CO2, in which “Target and Non-target” cells were separated by the two different CFSE concertation respectively. After the reaction, excess CFSE was quenched with equal volume of ice-cold FBS and subsequently washed in complete RPMI 1640. Lastly, 10 x 10^6^ cells/target/mouse (20 x 10^6^ cells) were given intravenously into control-vaccinated and p11-vaccinated mice. Twenty hours after adoptive transfer, the mice were sacrificed and spleens harvested and homogenized. Cells were stained for viability and fixed in 4% formaldehyde before acquisition on the BD LSRFortessa (BD Biosciences). The frequency of specific lysis was calculated by the ratio of %CFSE^high^ and %CFSE^low^ cells of p11-vaccinated mice compared to average of control mice: 100- [ (% CFSE_p11/CAF__09b_^high^/%CFSE_p11/CAF__09b_^low^cells)/($\bar{x}$ %CFSE_CAF__09b_^high^/%CFSE_CAF__09b_^low^cells) x 100 ].

#### Total IgG-ELISA

To measure humoral responses as performed previously in,^91^ Nunc MaxiSorp 96-well plates (Sigma-Aldrich) were coated with the CD8^+^ T cell peptides (JPT, Berlin, Germany) or CTH522 antigen (1 μg/ml) in carbonate buffer and incubated overnight at 4°C. For IgG antibody detection, plates were blocked with 1x PBS (prepared from 10x PBS, Gibco Invitrogen) containing 2% BSA for 2 h, followed by three washes with washing buffer (1x PBS +0.2% Tween). Serum samples were diluted in 1% BSA as specified in each figure and incubated overnight at 4°C. Following washing, samples were incubated at room temperature for 1 h with HRP-conjugated rabbit anti-mouse IgG (H + L) secondary antibody (AH Diagnostics). The reaction was developed using 3, 3′, 5, 5′-tetramethylbenzidine (TMB PLUS2, Kementec) for 5–15 min. The reaction was stopped with 0.5 M H2SO4 sulfuric acid (Honeywell Fluka). Absorbance was measured at 450 nm with background correction at 620 nm using a SunriseTM Absorbance Reader (Tecan Life).

#### Sandwich IFNγ ELISA

PBMCs and spleen single-cell suspensions were adjusted to 1 x 10^5^ cells/well in 96-well U-bottom plates (ThermoFisher) and cultured in the presence of 5 μg/mL antigen or peptides (JPT) for three days at 37°C and 5% CO2. Supernatants were collected from triplicate samples. To quantify IFN-γ concentrations, Nunc MaxiSorp 96-well plates (Sigma-Aldrich) were coated with rat anti-mouse IFN-γ (1:1000, BD Pharmingen, clone R46A2) in carbonate buffer (SSI Diagnostica) and incubated overnight at 4°C. Plates were blocked with 1x PBS containing 2% (w/v) skim milk powder (Natur Drogeriet) at room temperature for 2 h at. Diluted supernatants in PBS with 2% Bovine Serum Albumin (BSA) (Sigma-Aldrich) were added to the plates and incubated overnight at 4°C. For IFN-γ detection, samples were incubated with biotin rat anti-mouse IFN-γ (1:5000, BD Pharmingen, clone XMG1.2) in 1x PBS with 1% BSA at room temperature for 1 h. Subsequently, plates were incubated for 30 min with streptavidin-conjugated horseradish peroxidase (HRP) (BD Pharmingen, 1:5000) in 1x PBS with 1% BSA. Samples were developed as described above in the antibody ELISA procedure.

#### Immunohistochemical staining

Genital tracts were excised from infected mice post-euthanasia, fixed in 4% formaldehyde (VWR Chemicals) at room temperature, and paraffin-embedded for IHC analysis.

Subsequent processing, sectioning, and staining of the specimens were performed by technical staff at BioSiteHisto (Finland), as described elsewhere.^92^ Lastly, slides were digitalized as WSI in Mirax format using a 3DHistech Panoramic MIDI scanner (3DHistech Ltd.) and were visualized using Caseviewer version 2.3. Tissue sections immunohistochemically stained for CD4 and CD8 were assigned a cell infiltration score for CD8^+^ T cells. The following scores were used: 0; very few cells, sporadic, 1; very mild infiltration, sporadic, 2; mild infiltration, sporadic distribution, 3; moderate infiltration including 0–2 subepithelial clusters, 4; moderate infiltration, moderate numbers of subepithelial clusters (>2), 5; massive infiltration, massive numbers of supepithelial clusters (>10). All evaluations and scoring were performed completely blinded to mouse number, treatment group and infection status.

### Quantification and statistical analysis

All data analysis, graphs and plots were performed using GraphPad Prism v 9.3.1. Visual illustrations were created using BioRender under the publication license of Follmann, F. (2025) https://BioRender.com/7j3l8ks. The figure legends detail the specific statistical tests used and indicate *p*-values, which are shown either within the figures or in the accompanying legends. When data met the assumptions for parametric tests, these tests were used to assess significance. When the assumptions were not met, non-parametric tests were applied instead. Results with *p*-values exceeding 0.05 were considered not statistically significant, denoted as ns. The following *p*-value thresholds were applied: ∗*p* < 0.05, ∗∗*p* < 0.01, ∗∗∗*p* < 0.001, ∗∗∗∗*p* < 0.0001.
