# Supplementary material for: CD8+ T cell responses recognizing immunodominant Chlamydia antigens fail to protect against infection
Source: iScience. 2026 Mar 5;29(4):115242. doi: 10.1016/j.isci.2026.115242 (PMC13053786; doi:10.1016/j.isci.2026.115242)
Supplement: Document S1. Figures S1–S8 and Tables S1 and S2 [file mmc1.pdf]

iScience, Volume *29*

Supplemental information

CD8$^+$ T cell responses recognizing immunodominant *Chlamydia* antigens fail to protect against infection

Safia Guleed, Nina Dieu Nhien Tran Nguyen, Sharmila Subratheepam, Kristoffer Mazanti Melchiors, Anja Weinreich Olsen, Matias Ciancaglini, Anna Lena Kastner, Emanuele Nolfi, Daniel Pinschewer, Frank Follmann, Jan Pravsgaard Christensen, Alice Sijts, and Jes Dietrich

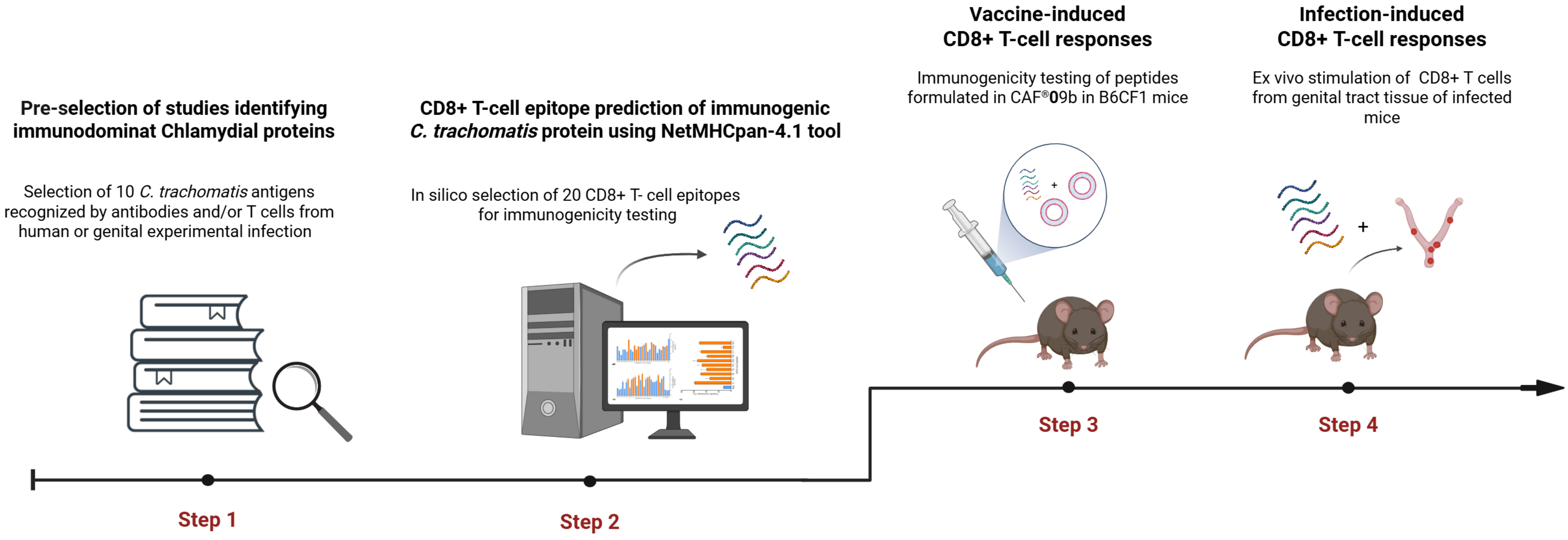

Figure S1. A in-silico approach for CD8+ T-cell epitope selection and discovery in immunogenic Chlamydia trachomatis proteins, related to Figure 1.

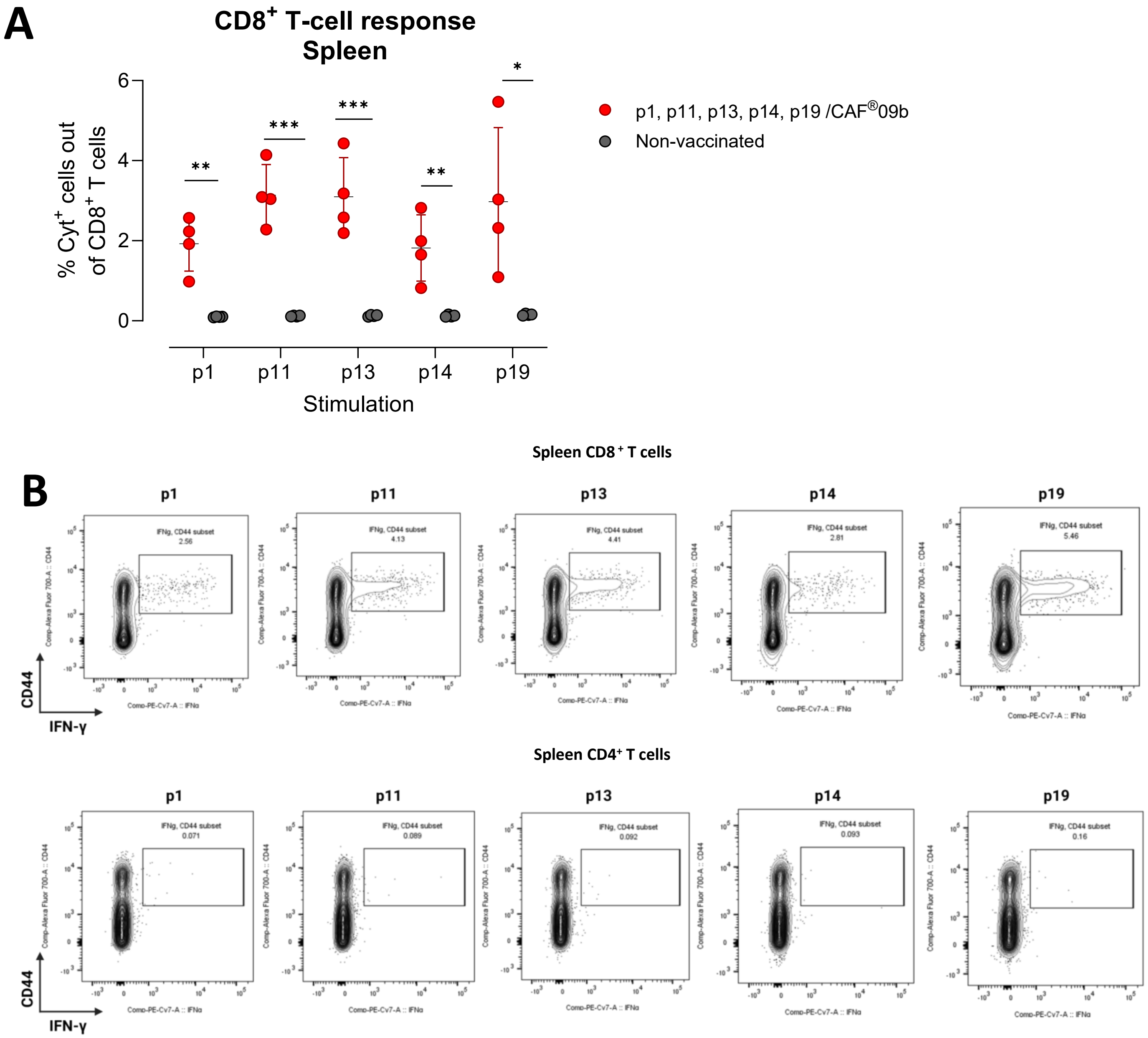

Figure S2. CD8+ T-cell responses after CAF®09b vaccination in B6C3F1 mice, related to Figure 2.

(A) p1, p11, p13, p14, p19/CAF®09b vaccinated and control mice (n = 8 biological replicates, pooled pairwise). Samples was stimulated with designated peptide (10 µg/ml) for 7 h. Antigen-specific responses are measured by flow cytometry with boolean-gating analysis of IFN-γ, TNFα, and/or IL-2 positivity (% Cyt+, mean ± SD, unpaired t-test).

(B) Representative contour plots over CD8+ and CD4+ T-cell responses.

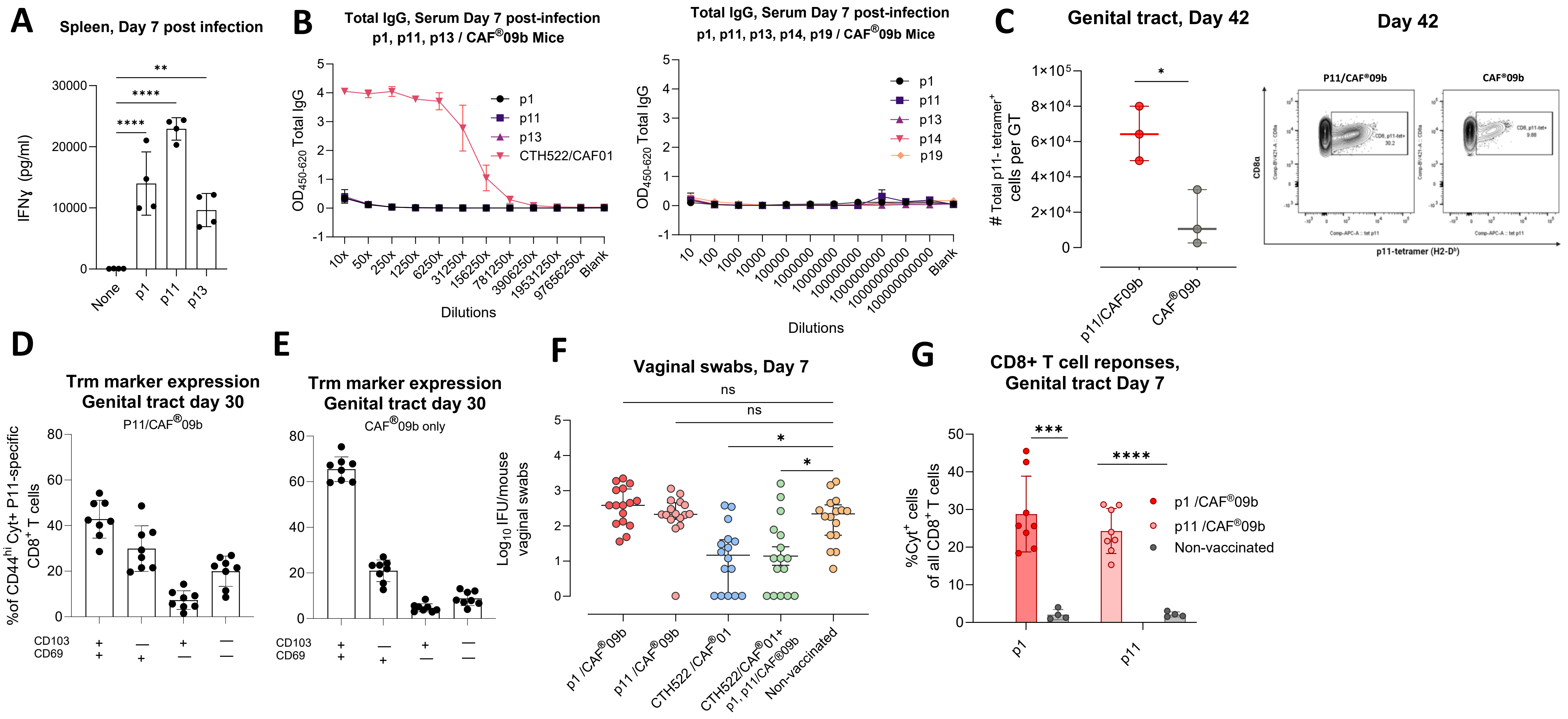

Figure S3. Post-infection responses after CAF®09b vaccination in B6CF31 mice, related to Figure 3.

Figure S3. Post-infection responses after CAF®09b vaccination in B6CF31 mice , related to Figure 3.

(A) IFN-γ release of splenocytes (n = 16 biological replicates, pooled pairwise) from p1, p11, 13/CAF®09b vaccinated mice measured by ELISA. Spleen cells were stimulated with designated peptide-antigens (5 µg/ml) for 72 h.

(B) Serum IgG antibody levels of p1, p11, 13/CAF®09b and CTH522/CAF®01 vaccinated mice (*left*) (n = 16 biological replicates, symbols = mean ± SD), and p1,11, 13, p14, p19/CAF®09b-vaccinated mice (*right*) (n = 8 biological replicates, symbols = mean ± SD).

(C) Absolute number of p11-tetramer+ in the GT of vaccinated mice day 42 post-infection (*left*) (n = 3 biological replicates, mean ± SD). Representative contour plot over p11-tetramer+ CD8+ T cells in the GT at Day 42 post infection (*right*) .

(D-E) Combination of Trm marker (CD69 and CD103) expression of $CD44^{hi}$ Cyt+ P11-specific CD8+ T cells present at day 30 post infection in the GT of (D) P11/ CAF®09b and (E) CAF®09b vaccinated groups (n = 8 biological replicates, mean ± SD).

(F) Bacterial burden at day 7 post infection of different vaccinated groups of animals (n = 16 biological replicates, median ± IQR, Kruskal-Wallis test with Dunn's multiple comparison test).

(G) Frequency Cyt+ CD8+ T cells in the GT of vaccinated mice (n = 16 biological replicates, pooled pairwise, mean ± SD, one-way ANOVA followed by Dunnett's multiple comparison test) day 7 post infection.

Cyt+ = Cytokine positive; IQR = interquartile range; GT= Genital tract; IFU = inclusion forming units. Statistical significance is represented by ns = non-significant, *p<0.05, **p<0.01, ***p<0.001, ****p<0.0001.

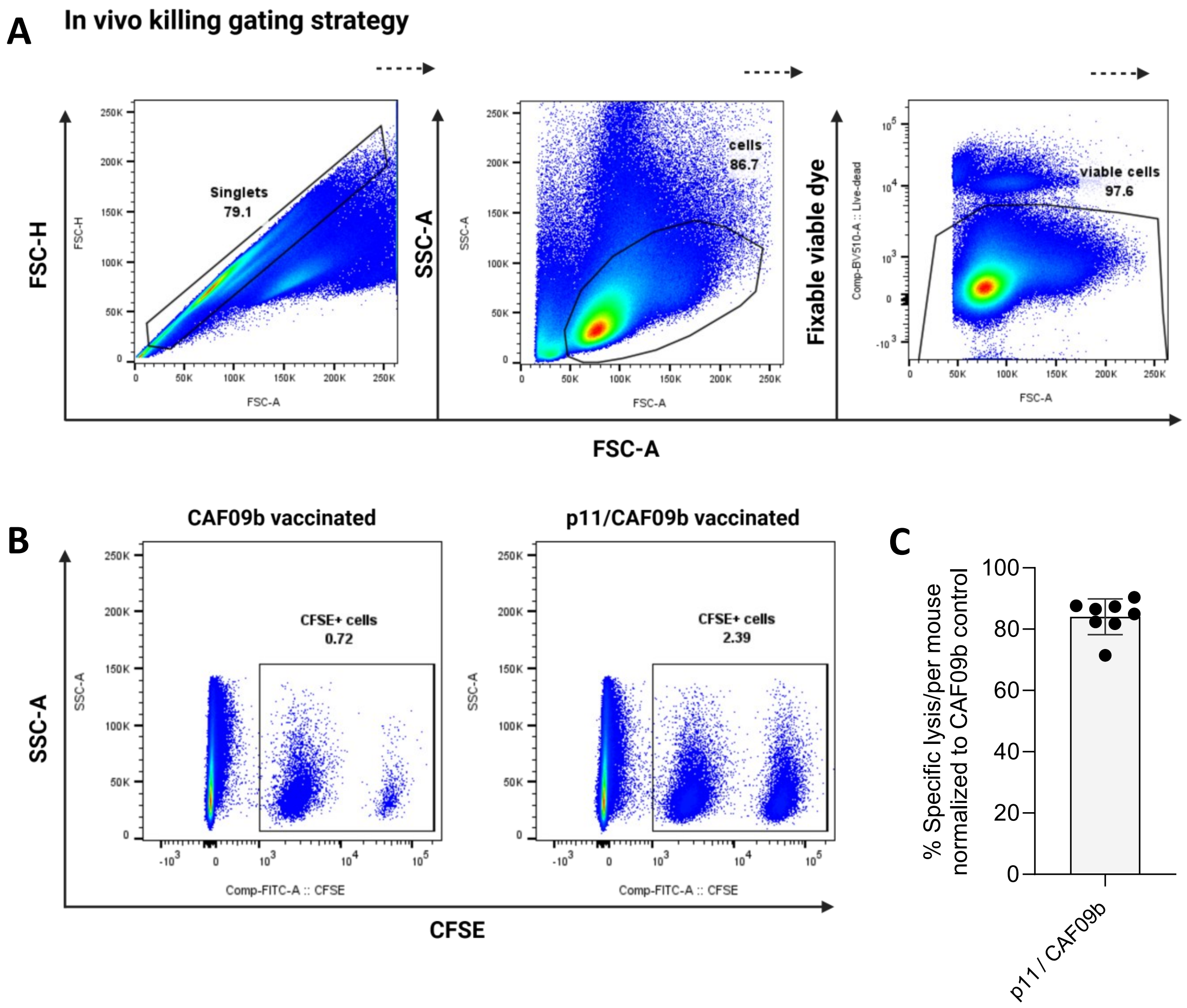

Figure S4. In vivo killing by CAF®09b-induced CD8+ T cells, related to Figure 3.

(A) Gating strategy to determine frequency of cytotoxic T lymphocyte killing.

(B) Representative flow cytometry dot plots of splenocytes from CAF®09b-vaccinated B6C3F1 mice intravenously injected with 20 x 10^6 cells CFSE labelled cells after 20 h of cell transfer.

(C) Quantification of percentage of specific lysis of "Target" p11-loaded splenocytes (CFSE^{high}) in p11/CAF®09b vaccinated mice (n = 8 biological replicates, mean ± SD).

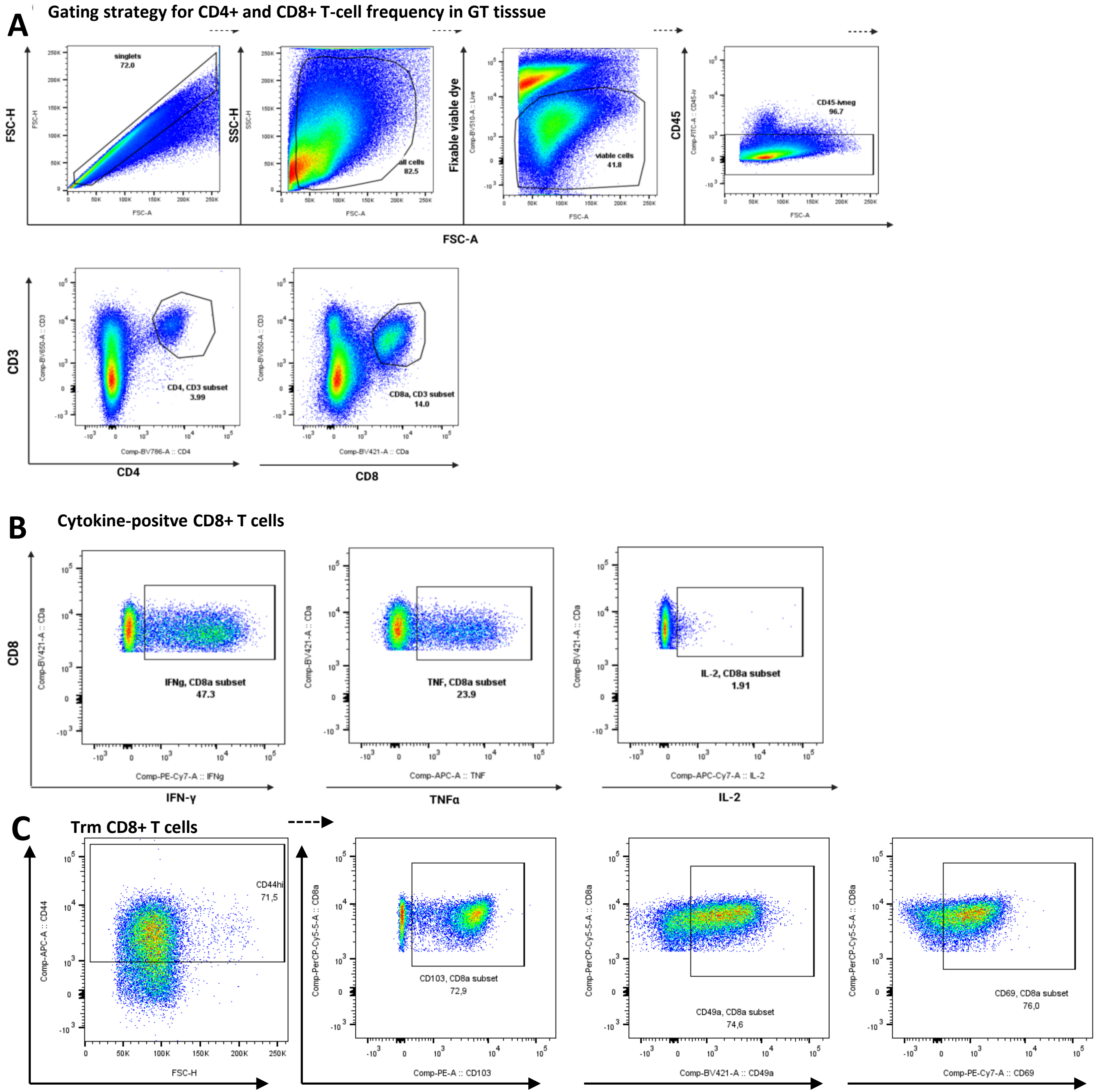

Figure S5. General gating strategies of T-cell immune responses in genital tract tissue cells, related to Figure 1-5.

(A) Discrimination of doublets, cell debris, dead cells and vascular leukocytes for identification of GT CD3+ CD4+ and CD3+ CD8+ cell frequencies.

(B) Gating of cytokine-positive CD8+ T cells (IFN-γ+, TNFα+, and/or IL-2+)

(C) Gating of CD44^{hi} CD8+ T cells followed by gating of Trm markers (CD103, CD49a, CD69)

GT = genital tract

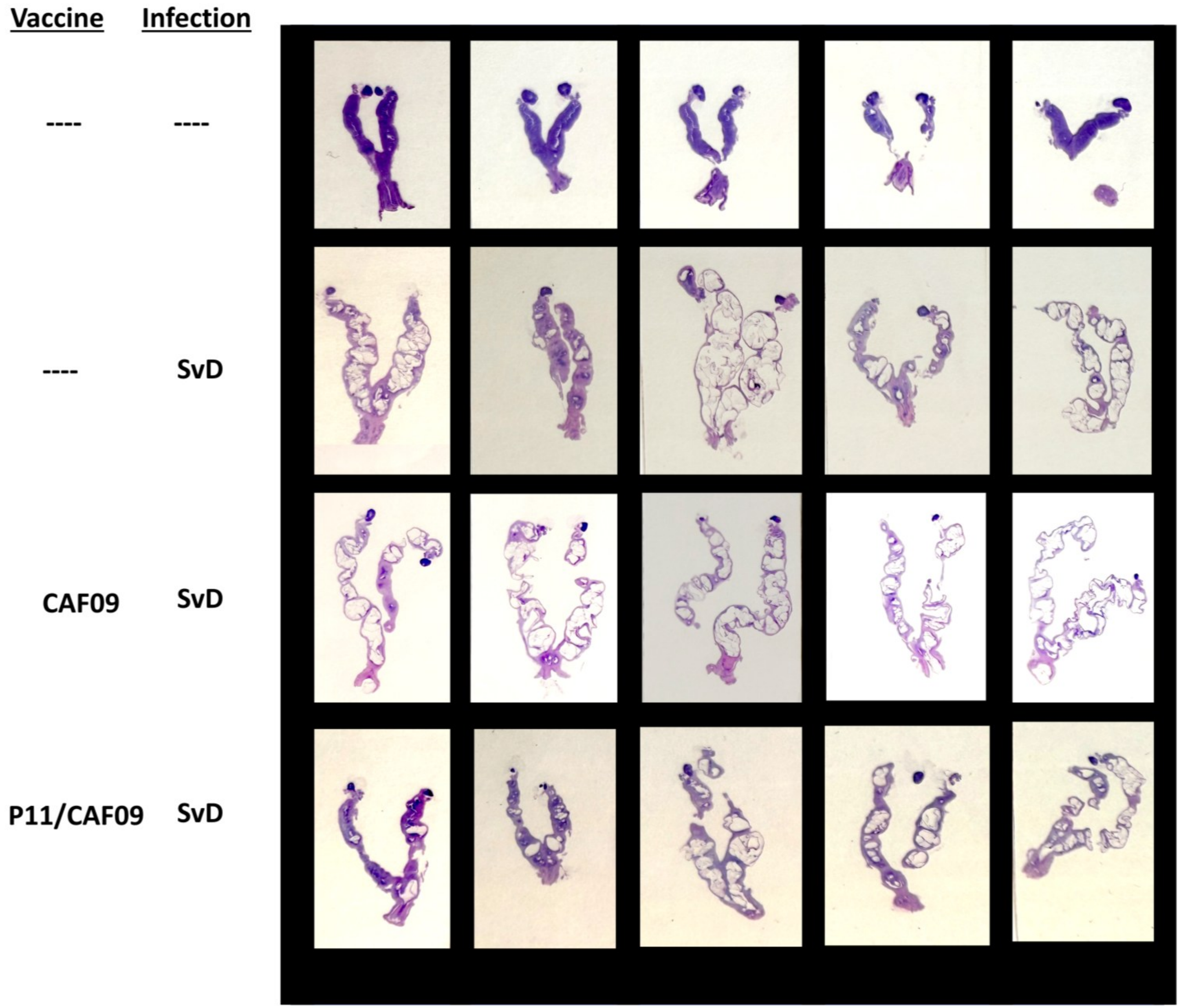

Figure S6. Histopathological changes post infection in groups of vaccinated and non-vaccinated mice, related to Figure 3. Groups of female B6C3F1 mice (n = 5 biological replicates) were vaccinated three times subcutaneously with P11/CAF®09 or CAF®09 with two weeks intervals. 4 weeks post immunization the mice received a transcervical infection with 10^3 IFUs of *C.t.* SvD. At day 50 post infection the genital tracts (GT) were excised, fixed and stained with hematoxylin and eosin (HE).

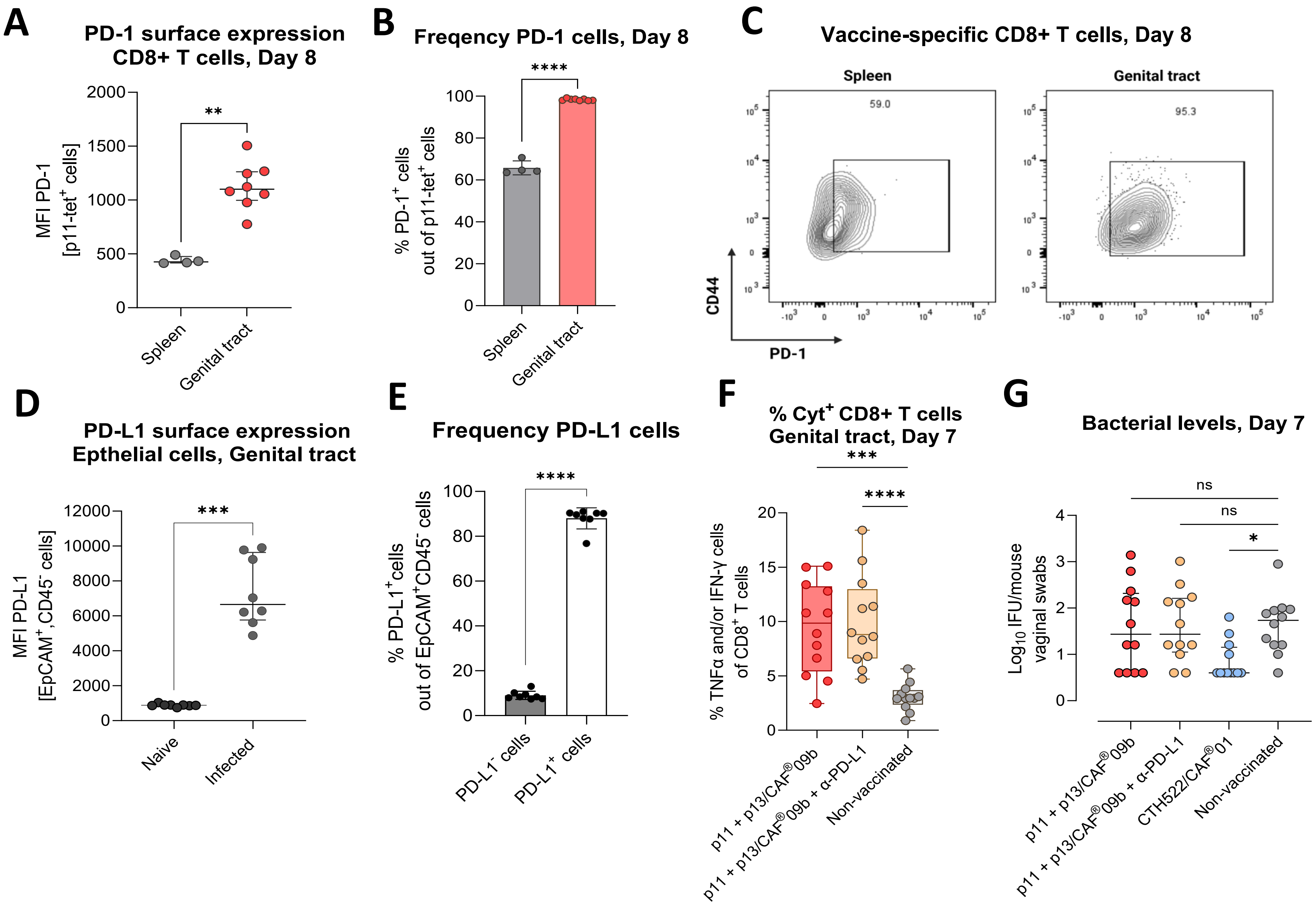

Figure S7. Effect of PD-L1 blockage on protection against *C. t.* infection, related to Figure 4.

Figure S7. Effect of PD-L1 blockage on protection against *C. t.* infection , related to Figure 4.

(A - C) Groups of female B6C3F1 mice (n = 8 biological replicates) were vaccinated i.p. three times during a two-week interval with p11 and p13 (30 μg/peptide) formulated in CAF®09b. Mice were vaginally challenged with *C. t.* (5 x 104 IFU) three weeks post final vaccination and organs collected at day 8 post infection.

(A) PD-1 expression on p11-tetramer specific (H2-Db) CD8+ T cells shown as median fluorescence intensity (MFI) in spleen (pair-wise pooled, c, median ± IQ, Mann-Whitney U test) and GT (median ± IQR, Mann-Whitney U test).

(B) Frequency of PD-1+ cells among p11-tetramer specific CD8+ T cells in the spleen (pair-wise pooled, mean ± SD, unpaired t-test) and GT (mean ± SD, unpaired t-test).

(C) Representative flow cytometry dot plots over p11-specific CD8 T cells in the spleen and GT expressing PD-1.

(D- E) MFI expression levels and frequency of PD-L1 on uterine epithelial cells (EpCAM+ and CD45.2-) of naïve or transcervically *C. t.* infected mice (n = 8 biological replicates, median ± IQ, Mann-Whitney U test) at day 7 post infection.

(F-G) Groups of female B6C3F1 mice (n = 12 biological replicates) were vaccinated as indicated with 30 μg per CD8+ T-cell peptide-antigen or 5 μg CTH522 formulated in CAF®09b or CAF®01 respectively. Mice were subjected to vaginal *C. t.* challenge and received i.p injections with anti-PD-L1 (200 μg) at day -2, 2 and 4 of infection.

(F) Flow cytometric frequency of TNFα+ and/or IFN-γ+ CD8+ T cells (mean ± SD, one-way ANOVA followed by Dunnett's multiple comparison test) in the GT.

(G) Enumerated bacterial levels (mean ± SD, Kruskal-Wallis test with Dunn's multiple comparison test) from vaginal swabs collected at day 7 post challenge.

i.p.= Intraperitoneal; CTH522 = *C. trachomatis* hybrid 522 antigen; Cyt+ = Cytokine positive; IFU = inclusion forming units; IQR = interquartile range; GT= Genital tract. Statistical significance is represented by ns = non-significant, *p<0.05, **p<0.01, ***p<0.001, ****p<0.0001.

A

In silico predicted CD8+ T-cell epitopes

Peptide No.	Peptide sequence	Peptide position	MHC I (H-2)
**p1**	EMFTNAACM	$MOMP^{D}_{117-125}$	**Db**
**p2**	SASFNLVGL	$MOMP^{D}_{150-158}$	**Db**
**p3**	NVLCNAAEF	$MOMP^{D}_{226-234}$	**Db**
**p4**	TAIFDTTTL	$MOMP^{D}_{311-319}$	**Db**
**p5**	AAHVNAQFRF	$MOMP^{D}_{384-393}$	**Db**
**p6**	RFDVFCTL	$MOMP^{D}_{132-139}$	**Kb**
**p7**	ASFQYAQS	$MOMP^{D}_{211-218}$	**Kb**
**p8**	LALSYRLNM	$MOMP^{D}_{275-283}$	**Kb**
**p9**	NMFTPYIGV	$MOMP^{D}_{282-290}$	**Kb**
**p10**	HEWQASLAL	$MOMP^{D}_{269-277}$	**Kk**
**p11**	TDTTFAWSV	$MOMP^{D}_{187-195}$	**Kk**

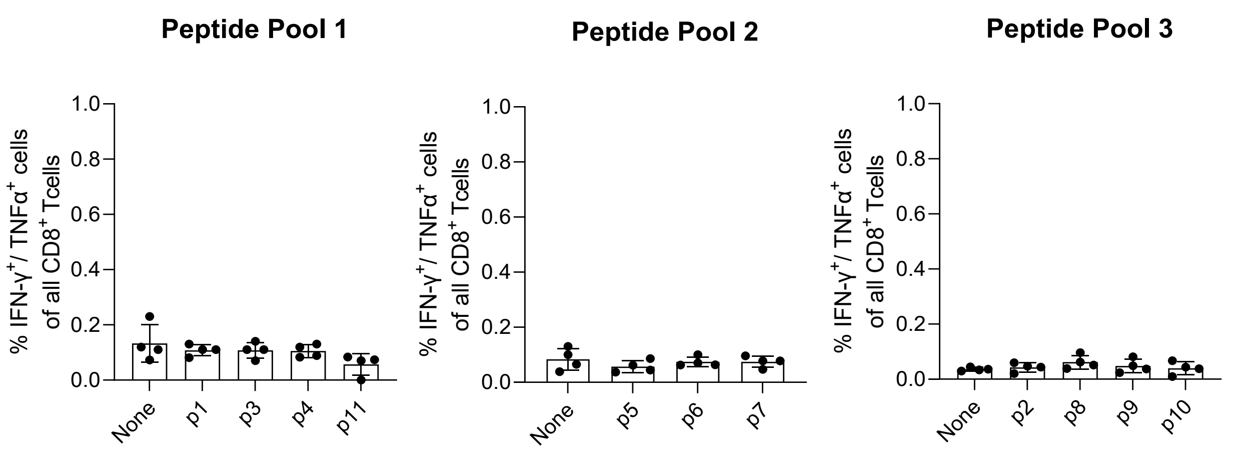

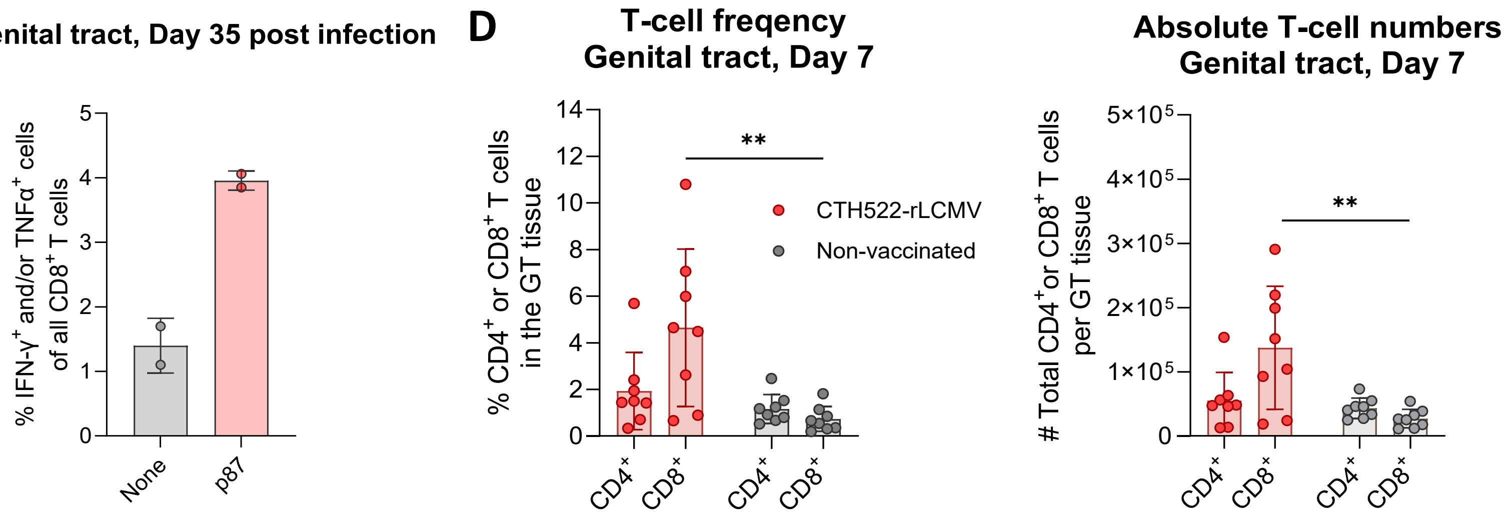

Figure S8. MOMP-specific CD8 T cell responses in BC63F1 mice, related to Figure 5.

Figure S8. MOMP-specific CD8⁺ T-cell responses in BC63F1 mice , related to Figure 5.

(A) Table of selected $CD8^+$ T-cell epitopes predicted from native MOMP SvD.

(B) Mice was vaccinated i.p. three times (n = 8 biological replicates, pooled pairwise), with two weeks interval, with peptide pools (15 µg/ peptide) consisting of designated peptides (Peptide pool 1, 2 and 3) formulated in CAF®09b. Spleens was harvested 10 days after final immunization and peptide-specific $CD8^+$ T-cell responses measured with flow cytometry of splenocytes re-stimulated with individual epitopes (10 µg/ml) from A.

(C) Genital tract cells from vaginally infected mice (n = 20 biological replicates, pooled in two) stimulated with or without the presence of p87 for 7 h. IFN-γ+ and/or TNFα+ CD8+ T-cell responses (mean ± SD) were measured by flow cytometry.

(D) CD8+ T-cell frequency (*left*) (mean ± SD, unpaired t-test) and numbers (*right*) (mean ± SD, unpaired t-test) in the genital tract of CTH522-rLCMV vaccinated or non-vaccinated mice following vaginal *C. t.* challenge.

C. t. protein	Peptide sequence	Peptide position*	Peptide No.	EL_Rank# (%)	BA_Rank# (%)	MHC class I (H-2)
**CT242 OmpH**	YQILNQSNL	89-97	p1	0.0043	0.0093	Db
	SVLLNEDIV	121-129	p2	0.0353	0.0284	Db
**CT443 OmcB**	ISVSNPGDL	296-304	p3	0.1768	0.2899	Db
	GQFTNNVVV	359-367	p4	0.0624	0.1013	Db
	VVFDSLPRL	454-462	p5	0.0154	0.0531	Kb
**CT442 CrpA**	ASFVNAIYL	61-69	p6	0.0012	0.0056	Db
**CT110 GroEL**	ISANNDAEI	148-156	p7	0.0811	0.0857	Db
	SANEGYDAL	473-481	p8	0.09	0.2222	Db
	HENMGAQMV	64-72	p9	0.0623	0.0973	Kk
	AESGRPLLI	239-247	p10	0.0368	0.1479	Kk
**CT111 GroES**	VQVGNIVLI	65-73	p11	0.0297	0.0538	Db
**CT541 Mip**	SSEKNKEPI	156-164	p12	0.301	0.2866	Db
**CT119 IncA**	IAPQITIVL	60-68	p13	0.0481	0.3455	Db
	ISLAGNALYL	74-82	p14	0.0626	0.0296	Db
	VSQDFYSCL	133-141	p15	0.0458	0.0383	Kb
**CT823 HtrA**	SALRNAISL	342-350	p16	0.001	0.0055	Db
**CT603 AhpC**	HAVINDLPL	136-144	p17	0.0082	0.0082	Db
	NDLPLGRSI	140-148	p18	0.0694	0.1383	Kk
**CT381 ArtJ**	VVLKDFPAL	170-178	p19	0.1274	0.1954	Kb
	EEIKHLVLV	95-103	p20	0.0285	0.1085	Kk

Table S1. List of selected and synthesized *Chlamydia trachomatis (C.t.)* CD8+ T-cell peptides predicted from proteins in Table 1, related to Figure 2.

* Amino acid 0 starts from mature part of the peptide sequence. # Selection based on natural Eluted ligand (EL) and binding affinity (BA) Rank (<0.5) = predicted as strong binder.

Peptide No.	Peptide sequence	Peptide No.	Peptide sequence	Peptide No.	Peptide sequence	Peptide No.	Peptide sequence
1	MKKLLKSVLVFAALS	36	FCTLGATSGYLKGNS	71	LALSYRLNMFTPYIG	106	QLNNMFTPYIGVKWS
2	LKSVLVFAALSSASS	37	GATSGYLKGNSASFN	72	YRLNMFTPYIGVKWS	107	MFTPYIGVKWSRASF
3	LVFAALSSASSLQAL	38	GYLKGNSASFNLVGL	73	MFTPYIGVKWSRASF	108	YIGVKWSRASFDSDT
4	ALSSASSLQALPVGN	39	GNSASFNLVGLFGDN	74	YIGVKWSRASFDADT	109	KWSRASFDSDTIRIA
5	ASSLQALPVGNPAEP	40	SFNLVGLFGDNENQK	75	KWSRASFDADTIRIA	110	ASFDSDTIRIAQPRL
6	QALPVGNPAEPSLMI	41	VGLFGDNENQKTVKA	76	ASFDADTIRIAQPKS	111	SDTIRIAQPRLVTPV
7	VGNPAEPSLMIDGIL	42	GDNENQKTVKAESVP	77	ADTIRIAQPKSATAI	112	RIAQPRLVTPVVDIT
8	AEPSLMIDGILWEGF	43	NQKTVKAESVPNMSF	78	RIAQPKSATAIFDTT	113	PRLVTPVVDITTLNP
9	LMIDGILWEGFGGDP	44	VKAESVPNMSFDQSV	79	PKSATAIFDTTTLNP	114	TPVVDITTLNPTIAG
10	GILWEGFGGDPCDPC	45	SVPNMSFDQSVVELY	80	TAIFDTTTLNPTIAG	115	DITTLNPTIAGCGSV
11	EGFGGDPCDPCATWC	46	MSFDQSVVELYTDTT	81	DTTTLNPTIAGAGDV	116	LNPTIAGCGSVAGAN
12	GDPCDPCATWCDAIS	47	QSVVELYTDTTFAWS	82	LNPTIAGAGDVKTGA	117	IAGCGSVAGANTEGQ
13	DPCATWCDAISMRVG	48	ELYTDTTFAWSVGAR	83	IAGAGDVKTGAEGQL	118	GSVAGANTEGQISDT
14	TWCDAISMRVGYYGD	49	DTTFAWSVGARAALW	84	GDVKTGAEGQLGDTM	119	GANTEGQISDTMQIV
15	MGDAISMRVGYYGDF	50	AWSVGARAALWECGC	85	TGAEGQLGDTMQIVS	120	EGQISDTMQIVSLQL
16	DAISMRVGYYGDFVF	51	GARAALWECGCATIL	86	GQLGDTMQIVSLQLN	121	SDTMQIVSLQLNNMF
17	MRVGYYGDFVFDRVL	52	ALWECGCATILGASF	87 (= 104)	DTMQIVSLQLNNMFT	122	QIVSLQLNNMFTPYI
18	YYGDFVFDRVLKTDV	53	CGCATILGASFQYAQ	88	IVSLQLNNMFTPYIG	123	LQLNNMFTPYIGVKW
19	FVFDRVLKTDVNKEF	54	TILGASFQYAQSKPK	89	QLNNMFTPYIGVKWS	124	NMFTPYIGVKWSRAS
20	RVLKTDVNKEFQMGA	55	ASFQYAQSKPKVEEL	90	MFTPYIGVKWSRASF	125	PYIGVKWSRASFDSN
21	TDVNKEFQMGAKPTT	56	YAQSKPKVEELNVLC	91	YIGVKWSRASFDADT	126	VKWSRASFDSNTIRI
22	KEFQMGAKPTTDTGN	57	KPKVEELNVLCNAAE	92	KWSRASFDADTIRIA	127	RASFDSNTIRIAQPK
23	MGAKPTTDTGNSAAP	58	EELNVLCNAAEFTIN	93	ASFDADTIRIAQPKS	128	DSNTIRIAQPKLAKP
24	PTTDTGNSAAPSTLT	59	VLCNAAEFTINKPKG	94	ADTIRIAQPKSATAI	129	IRIAQPKLAKPVVDI
25	TGNSAAPSTLTAREN	60	AAEFTINKPKGYVGK	95	RIAQPKSATAIFDTT	130	QPKLAKPVVDITTLN
26	AAPSTLTARENPAYG	61	TINKPKGYVGKEFPL	96	PKSATAIFDTTTLNP	131	AKPVVDITTLNPTIA
27	TLTARENPAYGRHMQ	62	PKGYVGKEFPLDLTA	97	TAIFDTTTLNPTIAG	132	VDITTLNPTIAGCGS
28	RENPAYGRHMQDAEM	63	VGKEFPLDLTAGTDA	98	DTTTLNPTIAGAGDV	133	TLNPTIAGCGSVVAA
29	AYGRHMQDAEMFTNA	64	FPLDLTAGTDAATGT	99	LNPTIAGAGDVKASA	134	TIAGCGSVVAANSEG
30	HMQDAEMFTNAACMA	65	LTAGTDAATGTKDAS	100	IAGAGDVKASAEGQL	135	CGSVVAANSEGQISD
31	AEMFTNAACMALNIW	66	TDAATGTKDASIDYH	101	GDVKASAEGQLGDTM	136	VAANSEGQISDTMQI
32	TNAACMALNIWDRFD	67	TGTKDASIDYHEWQA	102	ASAEGQLGDTMQIVS	137	SEGQISDTMQIVSLQ
33	CMALNIWDRFDVFCT	68	DASIDYHEWQASLAL	103	GQLGDTMQIVSLQLN	138	GQISDTMQIVSLQLN
34	NIWDRFDVFCTLGAT	69	DYHEWQASLALSYRL	104	DTMQIVSLQLNNMFT	139	KSVLVFAAL
35	RFDVFCTLGATSGYL	70	WQASLALSYRLNMFT	105	IVSLQLNNMFTPYIG	140	SVLVFAAL

Table S2. List of spCTH522 overlapping peptides, related to figure 5.

Peptides highlighted in red are the epitopes recognized by CD8+ T cells from rLCMV vaccinated or infected mice.
